# The role of S100A8 and S100A9 in external auditory canal cholesteatoma

**DOI:** 10.3389/fimmu.2024.1457163

**Published:** 2024-11-07

**Authors:** Guanwen He, Weijing Han, Zhongshou Zhu, Rifu Wei, Chang Lin

**Affiliations:** ^1^ Department of Otolaryngology, Head and Neck Surgery, The First Affiliated Hospital of Fujian Medical University, Fuzhou, Fujian, China; ^2^ Department of Otolaryngology, Ningde Municipal Hospital Affiliated of Ningde Normal University, Ningde, Fujian, China; ^3^ Department of Pathology, Heze Medical College, Heze, Shandong, China; ^4^ The School of Clinical Medicine, Fujian Medical University, Fuzhou, Fujian, China

**Keywords:** S100A8, S100A9, external auditory canal cholesteatoma (EACC), apoptosis, inflammatory, angiogenetic factors

## Abstract

**Background:**

Studies indicated that diverse cellular mechanisms including epithelial migration and hyper-proliferation, inflammatory responses, and enzymatic bone erosion were involved in the pathogenesis of cholesteatoma. S100A8 and S100A9, which are Ca2+-binding proteins belonging to the S100 family, can trigger the signaling pathways involved in the inflammatory processes, and a variety of cellular processes includes cell cycle progression, proliferation, and cell migration. However, the role of S100A8 and S100A9 and their associated inflammation and other signaling pathways in cholesteatoma have not been investigated yet. This study aimed to investigate the role of S100A8 and S100A9 in external auditory canal cholesteatoma and their potential pathological mechanisms.

**Methods:**

The study conducted histological staining, immunostaining, PCR, and Western blot to investigate the expression of S100A8/A9 and its related pathways in clinic EACC and the murine model of EACC.

**Results:**

Our data showed that there were increased mRNA and protein levels of S100A8 and S100A9 in clinical and animal models of EACC and the S100A8/A9 heterodimer protein was increased in the EACC model. Our study further demonstrated that the increased S100A8 and S100A9 were associated with apoptosis as well as inflammatory (TGF-β, IFN-γ, and IL-10) and angiogenetic (VEGF, HGF/SF, and c-Met) molecular pathways. The correlation analysis indicated that S100A8 and S100A9 were correlated with clinic staging, apoptosis, and inflammatory and angiogenetic factors.

**Conclusion:**

This study provided novel insight into the role of S100A8 and S100A9 associated with pathological mechanisms of EACC.

## Introduction

Cholesteatoma is a well-demarcated non-cancerous cystic lesion in the external or middle ear and mastoid characterized by the accumulation of keratinizing stratified squamous epithelium (1). It is non-neoplastic and composed of locally invasive masses with bone erosion (2, 3). External auditory canal cholesteatoma (EACC) is a rare form of cholesteatoma since the external auditory canal is an unusual site for cholesteatoma, which is usually in the middle ear or petrous apex (4). The overall incidence rate of EACC was around 0.30 per year per 100,000 people; comparably, the annual incidence of middle ear cholesteatoma is around 9.2 per 100,000 (5). EACC can occur in any age group, and the mean age of onset was 41.3 years (range 6–89 years; median 41 years) (6). Patients with EACC typically presented with otorrhea and chronic, dull pain due to the local invasion of squamous tissue into the bony external auditory canal (EAC) (7). Surgical removal is the main option for EACC treatment, but the rates of recurrence are high and challenge for patient management (8). Cholesteatoma’s aggressive growth properties, potential intracranial infections, and bone erosion have prompted scientific investigation of its diverse cellular mechanisms including epithelial migration and hyper-proliferation, inflammatory responses, and enzymatic bone erosion. Genome-wide microarray analysis provided broader, non-targeted molecular information on cholesteatoma and identified novel disease-associated molecular pathways. In a previous study, DNA chip analysis identified 104 upregulated genes in EACC compared with the control samples and the S100A8 and S100A9 were included (2), which indicated that S100A8 and S100A9 might play a role in pathogenies of EACC and required further investigation.

S100A8 and S100A9 belong to the S100 protein superfamily which contains a Ca2+-binding EF-hand domain with more than 20 members (9). S100A8 and S100A9 monomers can form stable non-covalently associated homodimers in the absence of calcium. In the presence of calcium, the heterodimer is the preferred form of human S100A8 and S100A9, which was also referred to as calprotectin with higher activity than the monomer (10). S100A8 and S100A9 are widely expressed and abundant in myeloid cells and were reported to be key factors in immune response and inflammation modulation. When the body is in a pathological state, such as inflammation and tumor, the S100A8 and S100A9 levels are abnormally elevated. S100A8 and S100A9 are released by activated granulocytes and act in a cytokine-like manner by binding to cell surface receptors (i.e., TLR4, RAGE, CD36, and CD11b/CD18) which trigger signaling pathways involved in the inflammatory pathway, and a variety of cellular processes includes cell cycle progression, proliferation, and cell migration. It has been shown that S100A8 and S100A9 can trigger many upregulated inflammatory mediators including TNF-α (11), interleukin (IL)-6, IL-10, TGF-β (12), chemokines, matrix metalloproteases (MMPs), and angiogenic factors VEGF (11, 13, 14). However, the role of S100A8 and S100A9 and their associated inflammatory and other signaling pathways in cholesteatoma have not been investigated yet. This study aimed to investigate the role of S100A8 and S100A9 and their potential pathological mechanisms in EACC.

The incidence rate of EACC was low and difficult to obtain clinical samples; therefore, the experimental animal models of this disease are of great significance for study. In this study, other than clinical samples, we built up an animal model of EACC to investigate the role of S100A8 and S100A9 and its potential pathological mechanisms in EACC.

## Method

### Patients and tissue samples

Cholesteatoma tissue samples were acquired from the external auditory canal of 30 subjects during lesion removal surgery between January 2018 and December 2020 in the Department of Otolaryngology in the author’s unit. The diagnosis of cholesteatoma was confirmed by histopathological examination. Small biopsy−acquired samples from the incision skin of 12 individuals undergoing tympanoplasty for chronic otitis media were utilized as the control group. The inclusion criteria were as follows: high-resolution CT scan of the temporal bone before treatment supported diagnosis of external auditory canal cholesteatoma. The exclusion criteria were as follows: incomplete medical records, the side of cholesteatoma was difficult to distinguish between external auditory canal and middle ear, and pathological examination does not support the diagnosis of cholesteatoma. The related information of clinical data was collected, as shown in **Table 1**. The study protocols were approved by the Research Ethics Committee of the Ningde Municipal Hospital Affiliated of Ningde Normal University (approved no. 20180602). All the participants were informed and provided written informed consent to participate and publish. These collected tissues were processed for histological staining, Western blot, and RT-qPCR.

**Table 1:** Correlation between S100A8 and S100A9 and clinic data.

		S100A8			S100A9		
n=30		Low (n=13)	High (n=17)	p	Low (n=15)	High (n=15)	p
Gender (%)	M	5 (38.46%)	6 (35.29%)	0.858	7 (46.67%)	4 (26.67%)	0.256
	F	8 (61.54%)	11 (64.71%)		8 (53.33%)	11 (73.33%)	
Age (years)		36.85 ± 20.46	35.41 ± 23.72	0.863	37.27 ± 23.18	34.80 ± 21.50	0.765
Lesion size (%)	L	6 (46.15%)	8 (47.06%)	0.125	7 (46.67%)	7 (46.67%)	0.513
	R	7 (53.85%)	5 (29.41%)		5 (33.33%)	7 (46.67%)	
	B	0 (0.00%)	4 (23.53%)		3 (20%)	1 (6.66%)	
Shin staging (%)	I	12 (92.31%)	8 (47.06%)	0.027*	13 (86.67%)	7 (46.67%)	0.0201*
	II/III	1 (7.69%)	9 (52.94%)		2 (13.33%)	8 (53.33%)	
Holt staging (%)	II	12 (92.31%)	10 (58.82%)	0.040*	14 (93.34%)	8 (53.33%)	0.0132*
	III	1 (7.69%)	7 (41.18%)		1 (6.66%)	7 (46.67%)	
Pain or earache (%)	Yes	11 (84.62%)	14 (82.35%)	1	12 (80%)	13 (86.67%)	0.624
	No	2 (15.38%)	3 (17.65%)		3 (20%)	2 (13.33%)	
Hearing loss congestion (%)	Yes	10 (76.92%)	17 (100.00%)	0.141	14 (93.34%)	13 (86.67%)	0.543
	No	3 (23.08%)	0 (0.00%)		1 (6.66%)	2 (13.33%)	
Tinnitus (n/%)	Yes	2 (15.38%)	2 (11.76%)	1	2 (13.33%)	2 (13.33%)	1
	No	11 (84.62%)	15 (88.24%)		13 (86.67%)	13 (86.67%)	
Ear pruritus (%)	Yes	1 (7.69%)	1 (5.88%)	1	1 (6.66%)	1 (6.66%)	1
	No	12 (92.31%)	16 (94.12%)		14 (93.34%)	14 (93.34%)	
Otorrhea, pus, bleeding (%)	Yes	3 (23.08%)	7 (41.18%)	0.515	7 (46.67%)	3 (20%)	0.121
	No	10 (76.92%)	10 (58.82%)		8 (53.33%)	12 (80%)	
Duration of symptoms (%)	<30 days	11 (84.62%)	11 (64.71%)	0.421	12 (80%)	10 (66.67%)	0.409
	≥30 days	2 (15.38%)	6 (35.29%)		3 (20%)	5 (33.33%)	
Bone destruction (%)	Sw	2 (15.38%)	3 (17.65%)	1	3 (20%)	2 (13.33%)	1
	Mw	11 (84.62%)	14 (82.35%)		12 (80%)	13 (86.67%)	

M, male; F, female; L, left side; R, right side; B, bilateral; Sw, single wall; Mw, multiwall.

### Experimental models of external auditory cholesteatomas in mice

External canal ligation was applied for the experimental model of EACC (15). The male and female mice weighing 25 g–30 g were purchased from Speifu Biotechnology Co., Ltd [Beijing, China; License No. SCXK (Jing) 2019-0010]. Animal protocols were approved by the Ethics Committee of Fujian Medical University (Approval No. FJMUIACUC2022-0081) on 25/12/2022. The process was followed with the guidelines of the animal ethics, and the number of experimental animals was reduced to a minimum. The C57BL/6 mice were anesthetized with 1.25% Avertin (0.6 mL/20 g; Sigma-Aldrich, St. Louis, MO, USA) by intraperitoneal injection. The mice underwent ligation of their right external auditory canals through intraarticular incisions with silk ligatures (6-0) around the cartilaginous canal. Three months after surgery, a micro-CT scan (Hiscan XM Micro CT with Hiscan Reconstruct and Analyzer software; Suzhou Hesfield Information Technology Co., Ltd, China) was performed to confirm the model’s success post 3-month surgery. There were 10 mice with the cholesteatoma tissues, and the tissues were collected and divided into different parts for HE staining, PCR, and Western blot. The four mice were subjected to mock experiments without external ear canal ligation as the control group.

### Hematoxylin–eosin staining

Tissues were fixated in 10% formaldehyde, embedded in paraffin, sliced in a rotational microtome to produce 3-micron slices, and then stained with hematoxylin–eosin for histological examination.

### TUNEL staining for apoptotic cells

Apoptosis was detected by DAB TUNEL (TdT-mediated dUTP Nick-End Labeling) Apoptosis Detection Kit (Servicebio, China). Paraffin-embedded sections were deparaffinized and dehydrated in graded alcohol and treated with proteinase K, 20 µg/mL, at room temperature for 15 min. Endogenous peroxidase activity was quenched with 3% hydrogen peroxidase in phosphate-buffered saline. After incubation with recombinant TdT enzyme/Biotin-dUTP for 1 h, slides were washed and incubated with streptavidin-HRP for 0.5 h and visualized with DAB and counterstained with hematoxylin. The number and percentage of apoptotic cells in the control and EACC were counted in three different areas of each section at a 200-fold magnification of the microscope. The percentages of apoptotic cells were calculated by apoptotic cell number (brown) dividing all cell numbers (blue). The density of TUNEL-positive cells in immunostaining was calculated by ImageJ (NIH, USA) software.

### Immunoblot analysis

The patient and mouse tissues were ground in liquid nitrogen with lysis buffer [50 mM Tris–HCl (pH 7.4), 100 mM NaCl, 1 mM DTT, 2 mM EDTA, 1% Triton X-100, and 0.1% SDS] supplemented with protease inhibitor cocktail. An equal amount of protein was separated on SDS-PAGE and then transferred to PVDF (Millipore, USA). The PVDF membranes were incubated with the primary antibodies overnight at 4°C as the following antibodies: anti-S100A8 (K002925P, Solarbio, China), anti-S100A9 (K006082P, Solarbio, China), anti-S100A8/A9 (NBP1-60157, Novus, China), anti-VEGFA (K106502P, Solarbio, China), anti-HGF/SF (A10, ADI-905-163-100, Enzo, USA), anti-MET (K007603P, Solarbio, China), anti-TGF beta 1 (EPR21143, ab215715, Abcam, USA), anti-IFN gamma Receptor beta/AF-1 (ab171081, Abcam, USA), anti-IL-10 (EPR1114, ab133575, Abcam, China), anti-β-actin (ab8226, USA). ECL (Millipore, USA) substrates were used to visualize protein bands.

### RT-qPCR

Total RNA was extracted from patient and mouse tissues using Neurozol reagent (MACHEREY-NAGEL, Germany), and cDNA was generated using a reverse transcription reagent kit (PROMEGA, USA). Real-time PCR was run with a SYBR Green PCR kit (Takara, China). β-Actin served as the internal control. qPCR analysis was performed on an ABI 7500 Real-Time PCR System (Applied Biosystems, Thermo Fisher Scientific, and USA) according to the instructions supplied by the manufacturer. The relative expression levels of the genes were calculated by comparing them with actin using the 2−ΔΔCT method. The primers were used as the following:

β-actin F TGACGTGGACATCCGCAAAG

β-actin R CTGGAAGGTGGACAGCGAGG

S100A8 F TGTCTCTGATGGCCTGAAGC

S100A8 R CCCTGTAGACGGCATGGAAA

S100A9 F CATGCTGATGGCGAGGCTAA\

S100A9 R CCACTGTGGTCTTAGGGGGT

VEGF F TCTACCGTCCGGGAATCCTT

VEGF R TACAACATCCGCCACAACGA

HGF/SF F TGCTTTGATTCTTTCAGCCCG

HGF/SF R CACTGTGAGCGCACGTTTTA

c-Met F TGGGCACCGAAAGATAAACCT

c-Met R ATCTGGGTGTTCCAGCACAG

TGF-β F GGGCTACCATGCCAACTTCT

TGF-β R GCACTTCAACAGTGCCCAAG

IFN-γ F ACTGTCGCCAGCAGCTAAAA

IFN-γ R GCTTAGGTTGGCTGCCTAGT

IL-10 F AGACAGACTTGCAAAAGAAGGC

IL-10 R TCGAAGCATGTTAGGCAGGTT

### Statistical analysis

Statistical analysis and graphic presentation were carried out with GraphPad Prism version 10.0 (GraphPad Software, USA) or SPSS version 23.0 (IBM. USA). The normality of data was test by the Shapiro–Wilk test. A Student’s t-test was used where the normality test passed; otherwise, the non-parametric Mann–Whitney test was used to analyze the data. Likewise, the Pearson or non-parametric Spearman methods were used for correlation analysis. The value of p < 0.05 is considered a significant difference.

## Result

### Pathological features of cholesteatoma

**Figure 1:**
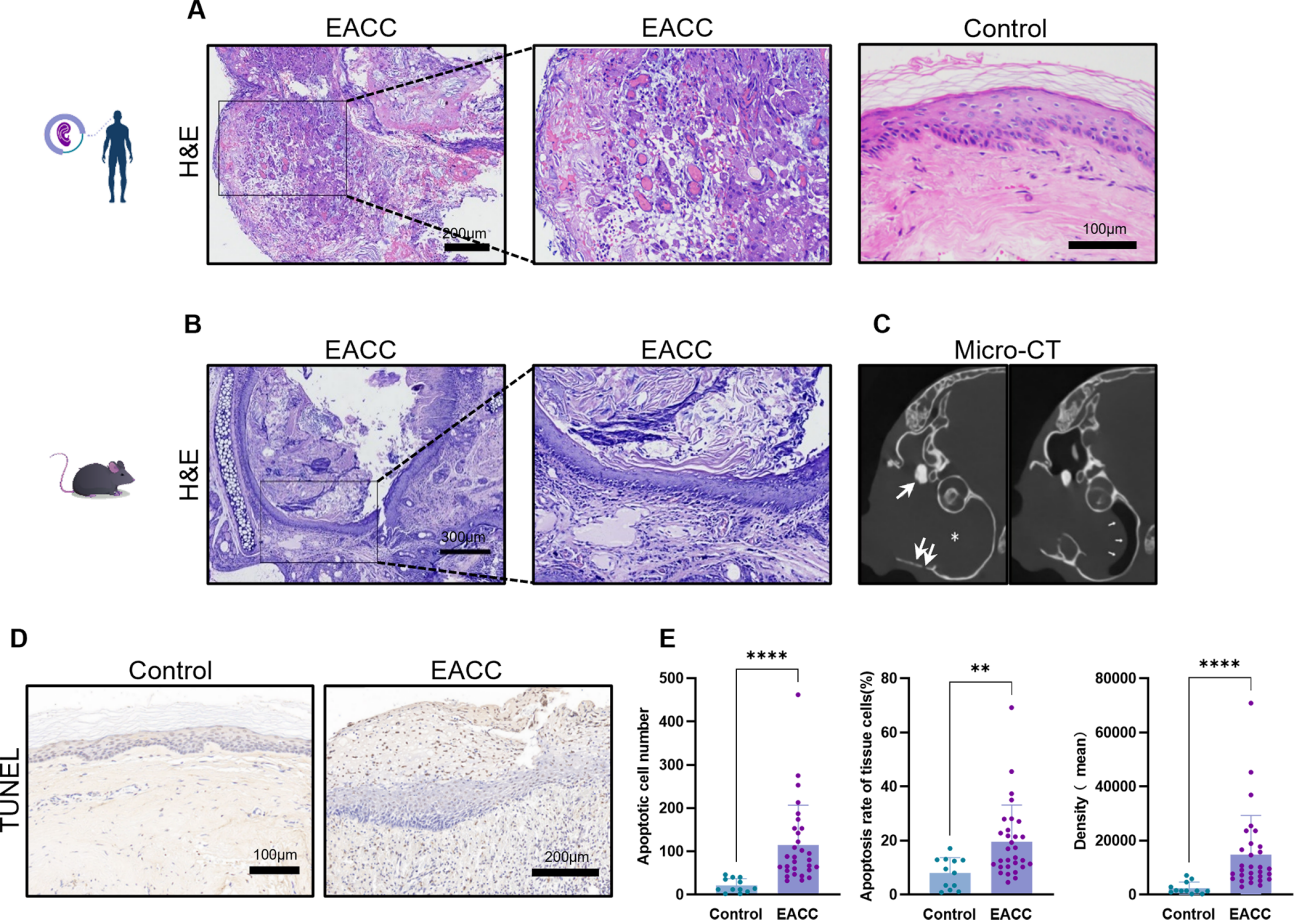
Pathological and imaging features of cholesteatoma. **(A)** The control and cholesteatoma tissues were processed to HE staining for the pathological staining. Representative images of EACC and the control were shown, and the scale bars were labeled. **(B)** Representative images for mouse EACC in HE staining were presented. **(C)** Representative images of CT scan for mouse EACC were presented. The shadow inside the drum indicated cholesteatoma invasion into the middle ear (star), with visible bone destruction of the tympanic wall and the auditory ossicle (thick arrow). The eardrum was compressed and deformed toward tympanum from the ear canal (thin arrow), indicating the formation of cholesteatoma in the external ear canal. **(D)** TUNEL staining was performed to detect the apoptotic cells in the control and patient EACC tissues. **(E)** The apoptosis was quantified by apoptotic cell number, apoptotic rate, and average intensity of TUNEL staining. **p< 0.01; ****p< 0.0001.

All the cholesteatoma diagnoses were confirmed by histopathological examination, and we first examined the pathological features of cholesteatoma. Compared with normal tissue (**Figure 1A**), the pathological features of cholesteatoma were shown by HE staining: destroyed normal epithelial structure, keratinized stratified squamous epithelium with granulation tissue, keratin debris, chronic inflammatory infiltrate and cholesterol clefts, the giant cells with foreign body, and congested blood vessels can be seen (**Figure 1A**).

To further investigate the pathogenesis of cholesteatoma, experimental models of EACC in mice were applied and the HE staining of animal model tissue also revealed a typical three-layer structure of cholesteatoma: keratinized fragments with inflammatory cell infiltration, keratinized squamous epithelium, and fibrous connective tissue of various thickness (**Figure 1B**). CT imaging showed that the external auditory canal and even including tympanic cavity was filled with soft tissue shadows and temporal bone destruction defects, indicating the formation of cholesteatoma, sometimes with the middle ear invasion (**Figure 1C**). TUNEL staining indicated that there were increased apoptotic cells (**Figure 1D**) which were quantified by number, rate, and density of TUNEL-stained cells (**Figure 1E**) in cholesteatoma tissues compared with the control.

### Increased mRNA and protein expression of S100A8 and S100A9 in EACC tissues

**Figure 2:**
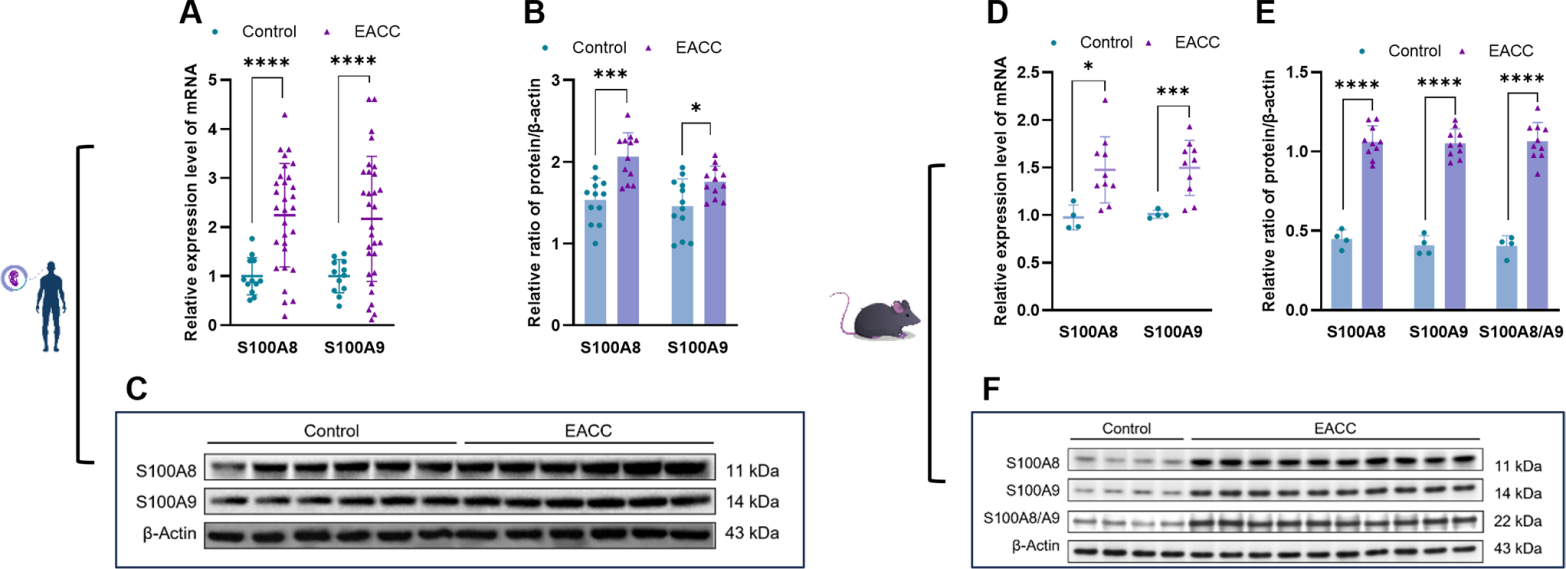
mRNA and protein expressions of S100A8 and S100A9 in EACC tissues. **(A)** qRT-PCR was determined for the relative expression levels of S100A8 and S100A9 in the control and patient EACC tissues. **(B)** Immunoblot analysis was applied to detect the protein levels of S100A8 and S100A9 in the control and patient EACC tissue with quantification by ImageJ. **(C)** The representative blot in **(B)** is presented. **(D)** qRT-PCR was determined for the relative expression levels of S100A8 and S100A9 in EACC mouse model tissues. **(E)** Immunoblot analysis was applied to detect the protein levels of S100A8, S100A9, and S100A 8/A9 in the control and mouse EACC tissue with quantification by ImageJ. **(F)** The representative blot in **(E)** is presented. *p< 0.05; ***p< 0.001; ****p< 0.0001.

The DNA chip analyses indicated that the S100A8 and S100A9 genes might be upregulated and involved in cholesteatoma pathogenesis. To examine the role of S100A8 and S100A9 in EACC, RT-qPCR was applied to determine the mRNA levels of S100A8 and S100A9 in the control and patient EACC tissue and it showed that both the mRNA levels of S100A8 and S100A9 were upregulated in EACC compared with the control (**Figure 2A**). To further confirm the expression, immunoblot was performed to detect the protein levels of S100A8 and S100A9 in the control and patient EACC tissue and it showed that the protein levels of S100A8 and S100A9 were upregulated in EACC compared with the control (**Figures 2B, C**). Similar results were shown in animal mode; both the mRNA levels of S100A8 and S100A9 were upregulated in the mouse EACC tissue compared with the control (**Figure 2D**). Immunoblot showed that the protein levels of S100A8, S100A9, and S100A8/A9 dimer were upregulated in the mouse EACC tissue compared with the control (**Figures 2E, F**). Notably, the level of the active form S100A8/A9 dimer was also significantly upregulated in mouse EACC. These data demonstrated that the mRNA and protein expressions of S100A8 and S100A9 were increased in both patient and mouse EACC tissues.

### S100A8 and S100A9 affected some inflammatory factors in EACC

**Figure 3:**
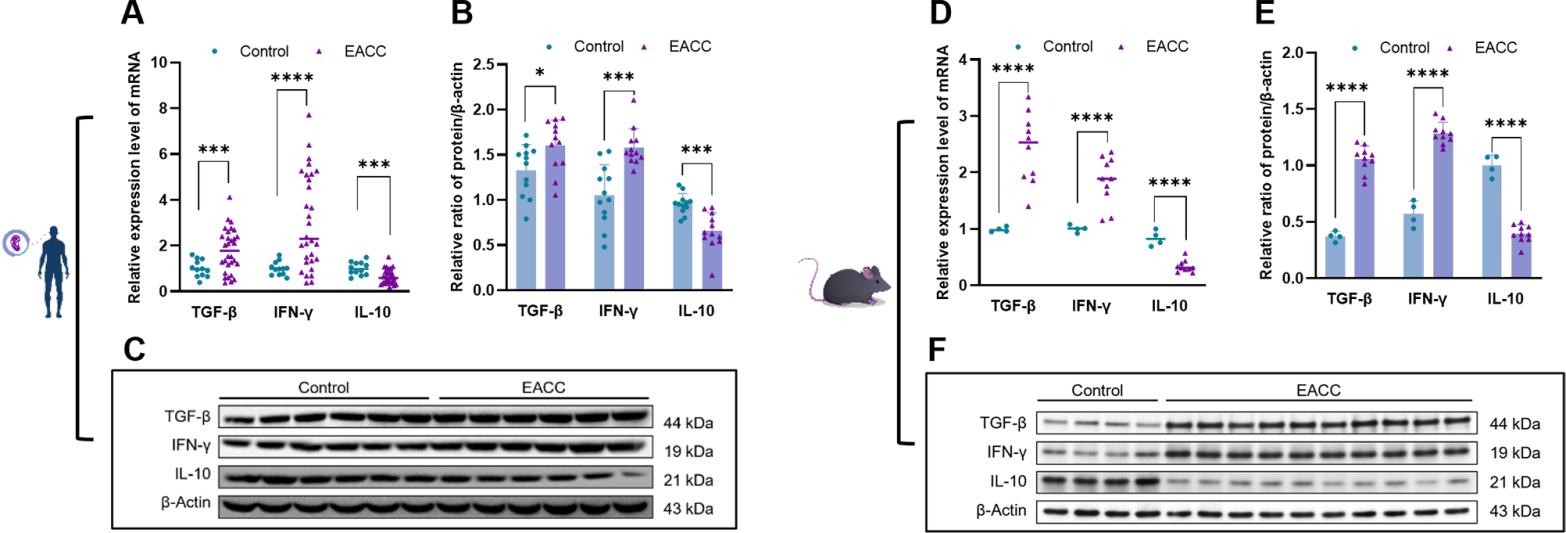
mRNA and protein expressions of inflammatory factors in EACC tissues. **(A)** qRT-PCR was determined for the relative expression levels of inflammatory factors TGF-β, IFN-γ, and IL-10 in the control and patient EACC tissue. **(B)** Immunoblot analysis was applied to detect the protein level of inflammatory factors TGF-β, IFN-γ, and IL-10 in the control and patient EACC tissue with quantification by ImageJ. **(C)** The representative blot in **(B)** is presented. **(D)** qRT-PCR determined the relative expression levels of inflammatory factors TGF-β, IFN-γ, and IL-10 in the control and mouse EACC tissues. **(E)** Immunoblot analysis was detected for the protein levels of inflammatory factors TGF-β, IFN-γ, and IL-10 in the control and mouse EACC tissues with quantification by ImageJ. **(F)** The representative blot in **(E)** is presented. *p< 0.05; ***p< 0.001; ****p< 0.0001.

Studies showed that inflammatory responses played a critical role in the pathogenesis of cholesteatoma (3). S100A8 and S100A9 play a critical role in inflammation, and we further investigate the role of S100A8- and S100A9-associated inflammation in patient EACC. RT-qPCR was applied to determine the mRNA levels of S100A8- and S100A9-associated inflammatory factors TGF-β, IFN-γ, and IL-10 gene in the control and patient EACC tissues, and it showed that the mRNA levels of TGF-β and IFN-γ were upregulated but IL-10 was downregulated in patient EACC compared with the control (**Figure 3A**). To further confirm the expression, immunoblot was performed to detect the protein levels of S100A8- and S100A9-associated inflammatory factors TGF-β, IFN-γ, and IL-10 in the control and EACC tissues, and the data demonstrated that the protein levels of TGF-β and IFN-γ were increased but the IL-10 level was decreased in the EACC group compared with the control (**Figures 3B, C**). Similar results were represented in mouse ECAA (animal model). In the mRNA level, it showed that TGF-β and IFN-γ were upregulated but IL-10 was downregulated in mouse EACC (**Figure 3D**). Immunoblot revealed that the protein levels of TGF-β and IFN-γ were increased but the IL-10 level was decreased in mouse EACC (**Figures 3E, F**). These data indicated that increased S100A8 and S100A9 were associated with inflammation in both clinic EACC and its animal model.

### S100A8 and S100A9 increased some angiogenetic factors in EACC

**Figure 4:**
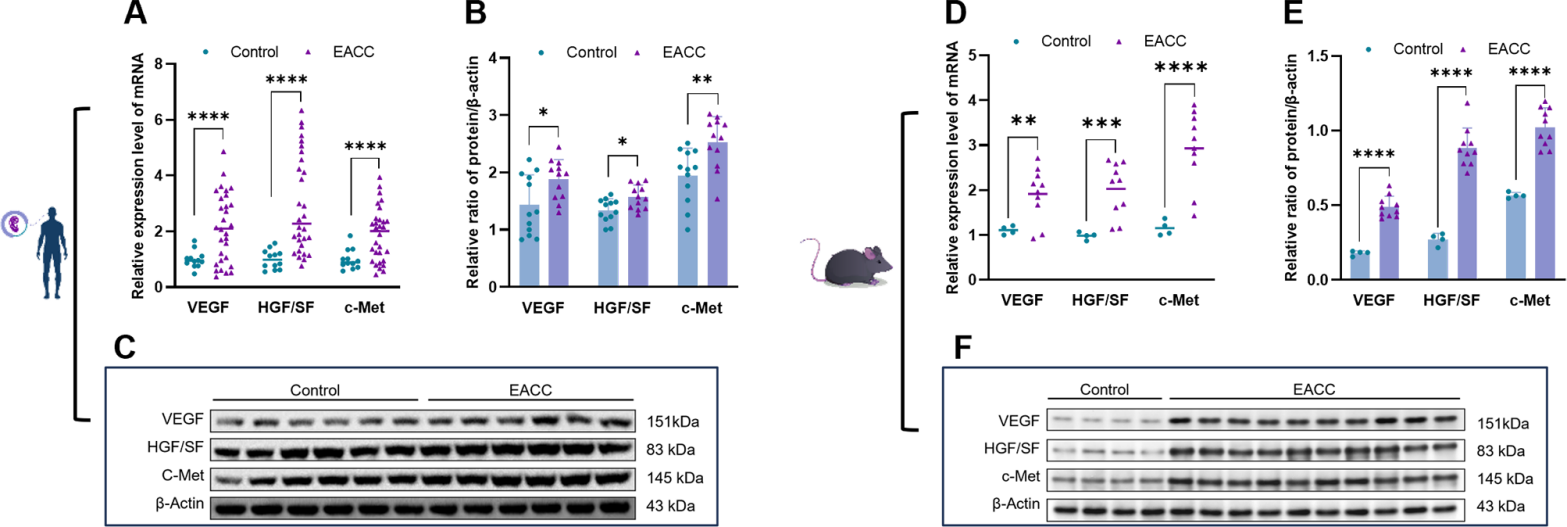
mRNA and protein expressions of angiogenetic factors in EACC tissues. **(A)** qRT-PCR was determined for the relative expression levels of the angiogenetic factors VEGF, HGF/SF, and c-Met in the control and patient EACC tissue. **(B)** Immunoblot analysis was applied to detect the protein levels of the angiogenetic factors VEGF, HGF/SF, and c-Met in the control and EACC tissue with quantification by ImageJ. **(C)** The representative blot in **(B)** is presented. **(D)** qRT-PCR was determined for the relative expression levels of the angiogenetic factors VEGF, HGF/SF, and c-Met in mouse EACC tissues. **(E)** Immunoblot analysis was applied to detect the protein level of the angiogenetic factors VEGF, HGF/SF, and c-Met in the control and mouse EACC tissue with quantification by ImageJ. **(F)** The representative blot in **(E)** is presented. *p< 0.05; **p< 0.01; ***p< 0.001; ****p< 0.0001.

Angiogenesis is involved in cholesteatoma and is associated with the severity of inflammation (16). We further investigated the role of S100A8- and S100A9-associated angiogenesis-related factors in EACC. RT-qPCR showed that the mRNA levels of the VEGF, HGF/SF, and c-Met were upregulated in patient EACC compared with the control (**Figure 4A**). Immunoblot demonstrated that the protein levels of the VEGF, HGF/SF, and c-Met were increased in patient EACC compared with the control (**Figures 4B, C**). In the animal model, both the mRNA (**Figure 4D**) and protein (**Figures 4E, F**) of the VEGF, HGF/SF, and c-Met were also increased in mouse EACC. These data indicated that S100A8 and S100A9 were associated with the dysregulation of the angiogenetic factors in clinical EACC and its animal model.

### The mRNA levels of S100A8 and S100A9 were correlated with clinical data, apoptosis, and some inflammatory and angiogenetic factors

**Figure 5:**
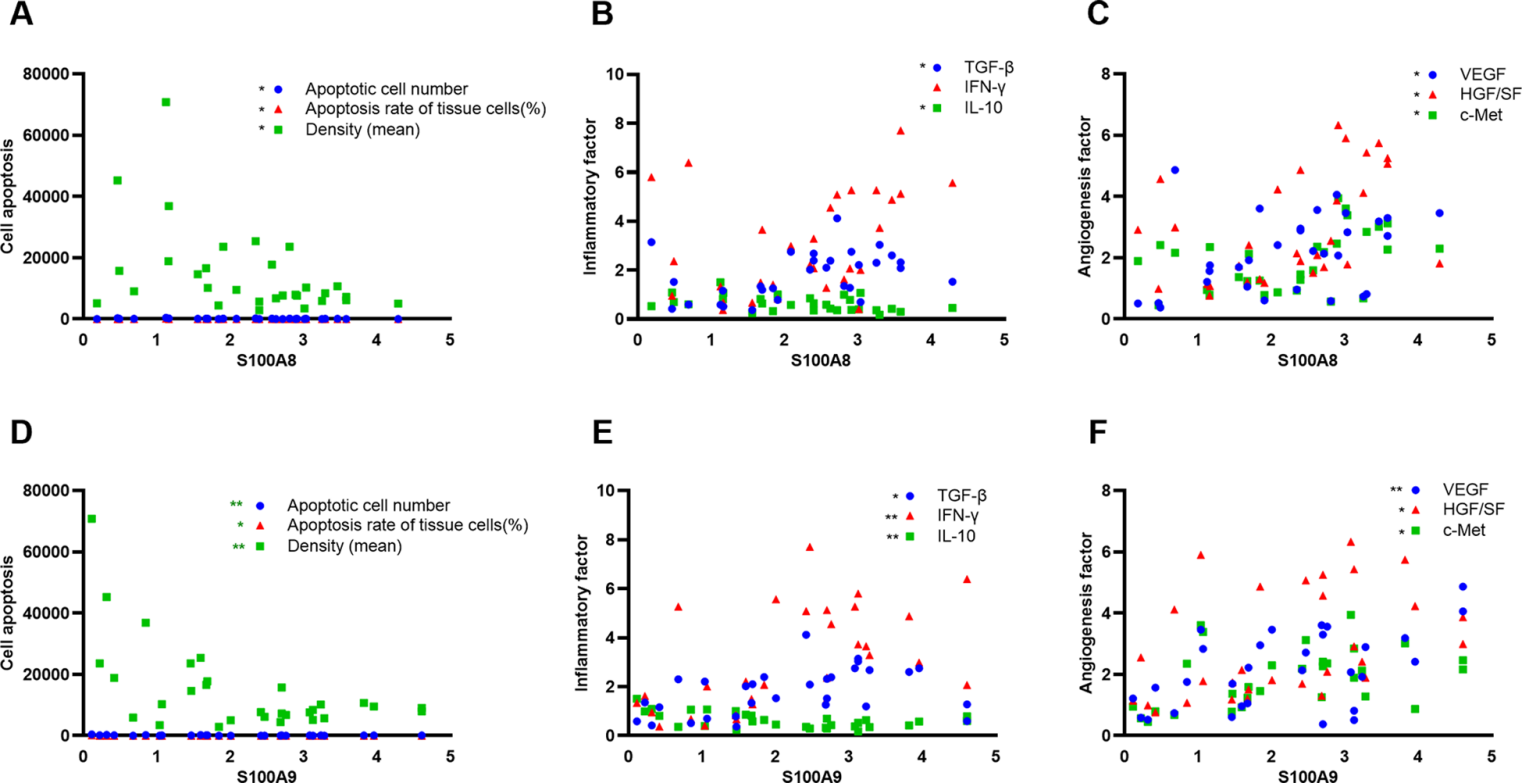
S100A8 and S100A9 were correlated with apoptosis, inflammation, and angiogenetic factors in clinical samples. **(A, D)** Correlation analysis between S100A8 **(A)** or S100A9 **(D)** and apoptotic index (apoptotic cell number, apoptotic rate, and average intensity of TUNEL staining) in clinical samples. **(B, E)** Correlation analysis between S100A8 **(B)** or S100A9 **(E)** and inflammatory factors TGF-β, IFN-γ, and IL-10. **(C, F)** Correlation analysis between S100A8 **(C)** or S100A9 **(F)** angiogenetic factors VEGF, HGF/SF, and c-Met.

To elucidate the role of S100A8 and S100A9 in clinical patients, we cut off the RT-qPCR optimal value for the mRNA levels of S100A8 and S100A9 as low or high groups and investigated the correlation between S100A8 and S100A9 and the clinical data. It indicated that the levels of S100A8 and S100A9 were correlated with the severity of EACC based on Shin staging (17) and Holt staging (18) classification, supporting the potential pathogenic factors of S100A8 and S100A9 in cholesteatoma bone erosion (**Table 1**). We further performed the correlation analysis and showed that the mRNA levels of S100A8 and S100A9 were both negatively correlated with apoptosis detected in the pathological examination (**Table 2**; **Figures 5A, D**). It also showed that the mRNA levels of S100A8 and S100A9 were positively correlated with the mRNA expression of the inflammatory factor TGF-β but negatively correlated with IL-10 (**Table 3**; **Figures 5B, E**). It also indicated that the mRNA levels of S100A8 and S100A9 were positively correlated with the mRNA expression of the angiogenetic factors VEGF, HGF/SF, and c-Met (**Table 3**; **Figures 5C, F**). These data supported that the mRNA levels of S100A8 and S100A9 were correlated with clinic data, apoptosis, and some inflammatory and angiogenetic factors.

**Table 2:** Correlation between S100A8 and S100A9 and apoptosis.

	Apoptotic cell number		The apoptosis rate of tissue cells (%)		Density (mean)	
	r	p	r	p	r	p
S100A8	−0.4226	0.02	−0.3822	0.0372	−0.4537	0.0118
S100A9	−0.5712	0.001	−0.4082	0.0251	−0.5689	0.001

**Table 3:** Correlation between S100A8 and S100A9 and inflammatory/angiogenetic factor expression in clinic ECAA.

	TGF-β		IFN-γ		IL-10		VEGF		HGF/SF		c-Met	
	r	p	r	p	r	p	r	p	r	p	r	p
S100A8	0.411	0.024	0.353	0.055	-0.45	0.013	0.374	0.042	0.442	0.014	0.406	0.03
S100A9	0.388	0.034	0.546	0.002	-0.51	0.004	0.527	0.003	0.437	0.016	0.401	0.03

## Discussion

In this study, increased mRNA and protein of S100A8 and S100A9 were found in the tissues of clinical and mouse models of EACC and S100A8/A9 dimer was increased in mouse EACC. Our data also showed that both mRNA and protein levels of S100A8 and S100A9 were associated with the increased inflammatory factors (TGF-β and IFN-γ) and angiogenesis-related factors (c-Met, HGF/SF, and VEGF) but decreased IL-10 in both patient and mouse EACC tissues. Correlation analysis indicated that S100A8 and S100A9 were significantly correlated with clinical staging, apoptosis, and inflammatory and angiogenetic factors. Notably, S100A8 and S100A9 are especially related to the severity of bone destruction caused by cholesteatoma based on Shin staging and Holt staging classification. Our study provided a novel insight into the pathological mechanisms of S100A8- and S100A9-associated apoptosis, inflammation, and angiogenesis.

Interestingly, the studies showed that keratin debris was cumulated as a result of an increase in the percentage of dead cells with apoptosis in cholesteatoma (19). Olszewska et al. (20) applied *in situ* TUNEL staining technology to show that the proportion of apoptotic cells in cholesteatoma epithelium was significantly higher than that in normal epidermal tissue, indicating that apoptosis played an important role in the pathogenesis of cholesteatoma. Our data showed that increased apoptotic cells in EACC were paradoxically negatively associated with S100A8 and S100A9. At the same time, S100A8 and S100A9 were positively correlated with inflammatory (TGF-β) and angiogenetic factors (VEGF, HGF/SF, and c-Met). S100A8 and S100A9 mainly induce pro-inflammatory and pro-tumor signaling pathways, and it is hard to explain the correlation with apoptosis in this context. Although the apoptosis increased in EACC, S100A8 and S100A9 might still play a more prominent role in cell proliferation and pro-tumor function, which can explain its negative correlation with apoptosis. The apoptotic cell is mainly involved in keratinocytes in cholesteatoma epithelium (20). The apoptotic mechanism resulted in that the cholesteatoma epithelium did not trigger uncontrolled proliferation of the cells and were different from other malignant epithelial tumors (21).

The pathological feature of EACC included that there were plenty of chronic inflammatory cells in granulation tissue (22) (**Figures 1A, B**), indicating that inflammation plays a critical role in cholesteatoma pathogenesis. S100A8 and S100A9 were found increased in inflammatory diseases and cancers (23) including osteosarcoma (24), lung carcinoma (25), and cervical cancer (26). Jonsson et al. showed that the blood levels of S100A8 and S100A9 were significantly elevated and positively correlated with the severity of sepsis. Plasma S100A8 and S100A9 had high specificity and sensitivity in predicting infection (27). Recent studies have also shown that upregulated S100A8 and S100A9 and their associated inflammatory factors were presented in the tumor microenvironment since the inflammation conditions are strongly involved in the neoplastic process and cancer development (11). Our data indicated the increased S100A8 and S100A9 were associated with increased IFN-γ and TGF-β but decreased IL-10 in clinical and animal models of EACC, consistent with the pathogenesis mechanism of inflammation involved in cholesteatoma pathogenesis. S100A8 and S100A9 band to the immune receptors and initialed the inflammatory signaling pathway, supporting their critical role in inflammation. Interestingly, the principal function of IL-10 was thought to be an anti-inflammatory cytokine and limit the immune response (28). S100A8 and S100A9 were negatively associated with decreased IL-10, consistent with the pro-inflammatory function of S100A8 and S100A9. Notably, we applied an animal model to support this study. The low incidence in EACC led to the difficulty in obtaining clinical specimens; therefore, animal models can make up for the defects. This study confirmed that external ear canal ligation in mice was a simple, feasible, and reliable method for constructing an animal model for EACC. After external ear canal ligation, local inflammatory reactions stimulated the skin to undergo squamous epithelial metaplasia and fibrosis, resulting in the formation of cholesteatoma.

Angiogenesis is crucial to the supply of a growing cell including the tumor cells with nutrition and oxygen. Angiogenesis appears in the subepithelial connective tissue of cholesteatoma (perimatrix), which sustains the proliferation migration of keratinocytes and the expansion of cholesteatoma. There was multiple evidence that angiogenesis was involved in cholesteatoma and associated with the severity of inflammation (22, 29). Angiogenesis is influenced by a large number of angiogenic factors including VEGF, HGF/SF, and its receptor c-Met (29, 30). Our data showed that increased VEGF, HGF/SF, and c-Met were associated with increased S100A8 and S100A9 in EACC, supporting the role of angiogenesis in EACC.

**Figure 6:**
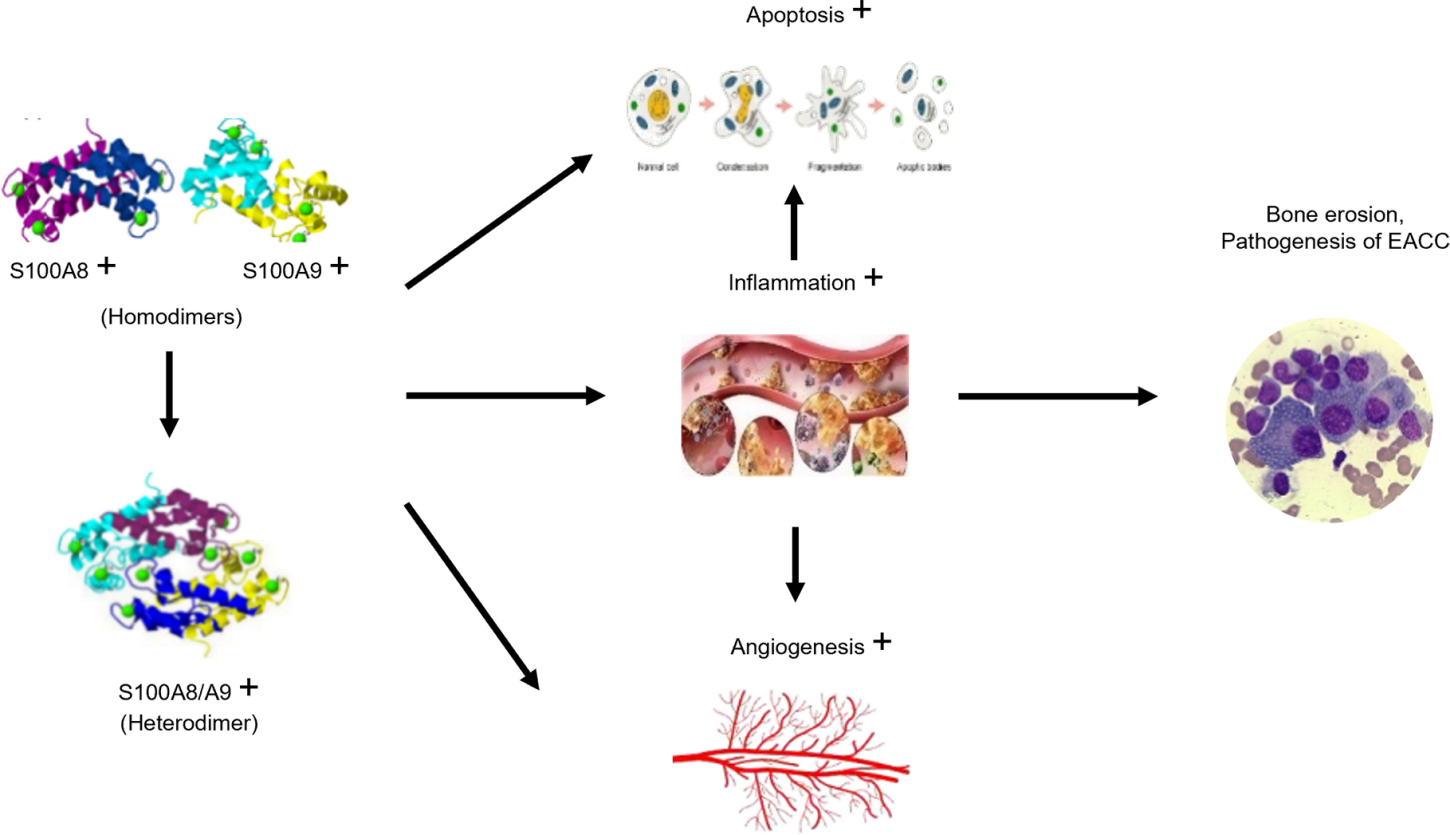
Schematic diagram of the proposed mechanism for S100A8 and S100A9 in EACC. The increased S100A8 and S100A9 band together and formed the S100A8/S100A9 heterodimer, which trigger signaling pathways associated with apoptosis, inflammation, and angiogenesis. Inflammation also promoted apoptosis and angiogenesis, finally leading to the pathogenesis, such as bone erosion in EACC.

In this study, our data demonstrated that the increased S100A8 and S100A9 were associated with increased apoptosis, inflammation, and angiogenesis in clinical and mouse models of EACC. These three factors (apoptosis, inflammation, and angiogenesis) may interact with each other (**Figure 6**), and inflammation also affected apoptosis and angiogenesis. For example, upregulated TGF-β in EACC is involved in cell proliferation, differentiation, and apoptosis. TGF-β promoted the apoptosis of cholesteatoma epithelial cells and inhibited the high proliferative capacity of cholesteatoma epithelium, making cholesteatoma not infinitely proliferate like malignant tumors (30).TGF-β also had effects on angiogenesis. There was evidence that TGF-β stimulated the production of VEGF and promoted angiogenesis. A study revealed decreased microvessel formation in the presence of IL-10 (31). Another study revealed that there was increased angiogenesis in an IL-10 KO mouse model (32). These studies support that IL-10 had anti-angiogenic effects. In this study, the decreased IL-10 might contribute to angiogenesis and the pathogenesis of EACC. In response to the inflammatory mediators, the endothelial cells promote angiogenesis. The endothelial cells proliferate and migrate to form new capillaries (angiogenesis), restoring nutrient levels and facilitating immune cell migration and triggering a vicious cycle (33). These indicated that apoptosis, inflammation, and angiogenesis interact with each other and this pathway network was associated with the EACC pathogenesis, such as bone erosion.

With recent advances, the current understanding of cholesteatoma pathogenesis has deepened into cellular and molecular levels. Genetic and epigenetic factors have been found to be involved in the progress of cholesteatoma. Some novel candidate genes and microRNA were identified for cholesteatoma via both genetic variants from exome sequence data and DEGs from mRNA-seq using cholesteatoma and middle ear mucosal tissues.

The results of these studies are expected to reveal a novel mechanism of cholesteatoma pathogenesis (34, 35). Employing the mass spectrometry-based proteomics and bioinformatics, another study demonstrated that SNCA overexpression in cholesteatoma might maintain the proliferative ability of cholesteatoma keratinocytes by promoting autophagy under inflammatory conditions (36).

## Conclusion

Our study showed that S100A and S100A9 increased in clinical and mouse models of EACC and were associated with apoptosis as well as inflammatory (TGF-β, IFN-γ, and IL-10) and angiogenetic (VEGF, HGF/SF, and c-Met) molecular pathways. The correlation analysis supported that S100A8 and S100A9 were correlated with clinical staging, apoptosis, and inflammatory and angiogenetic factors. This study provided novel insight into the role of S100A8 and S100A9 associated with pathological mechanisms in EACC.

## Data Availability

The original contributions presented in the study are included in the article/**Supplementary Material**. Further inquiries can be directed to the corresponding author.
