# Supplementary figures and images for: The role of S100A8 and S100A9 in external auditory canal cholesteatoma
Source: Front Immunol. 2024 Nov 7;15:1457163. doi: 10.3389/fimmu.2024.1457163 (PMC11578731; doi:10.3389/fimmu.2024.1457163)

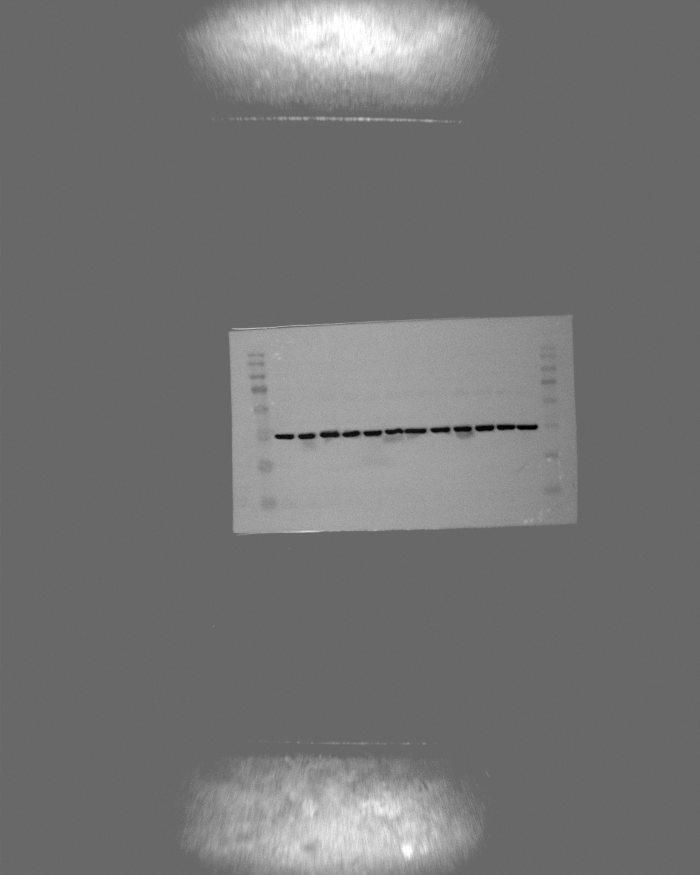

Supplement: Supplementary file 1 [file DataSheet1.zip › original-imagesú¿╤╣╦⌡║≤ú⌐/human blot/1/actin(control 1-6+EACC 1-6).tif]

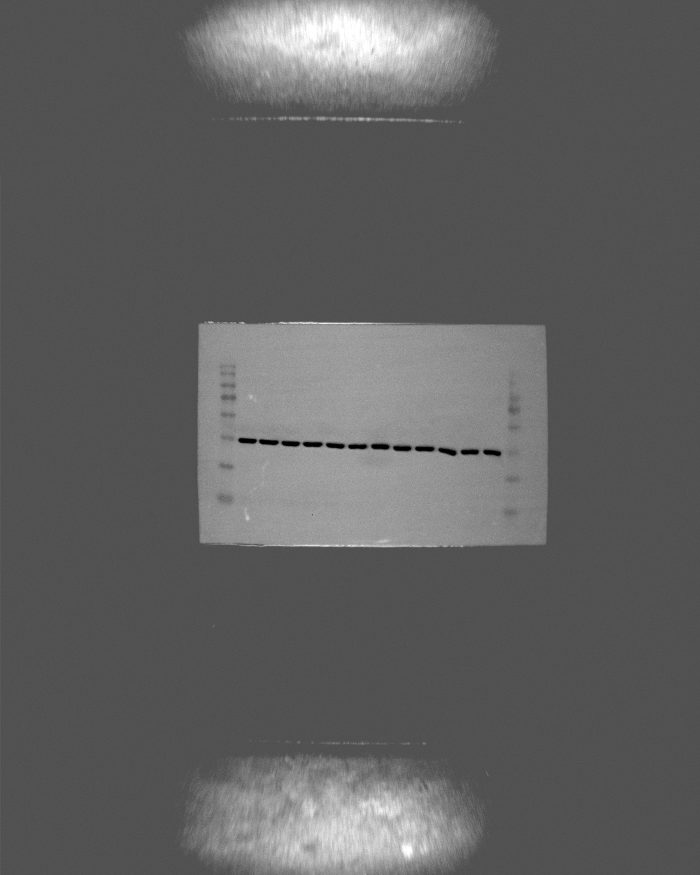

Supplement: Supplementary file 1 [file DataSheet1.zip › original-imagesú¿╤╣╦⌡║≤ú⌐/human blot/1/actin(control 7-12+EACC 7-12).tif]

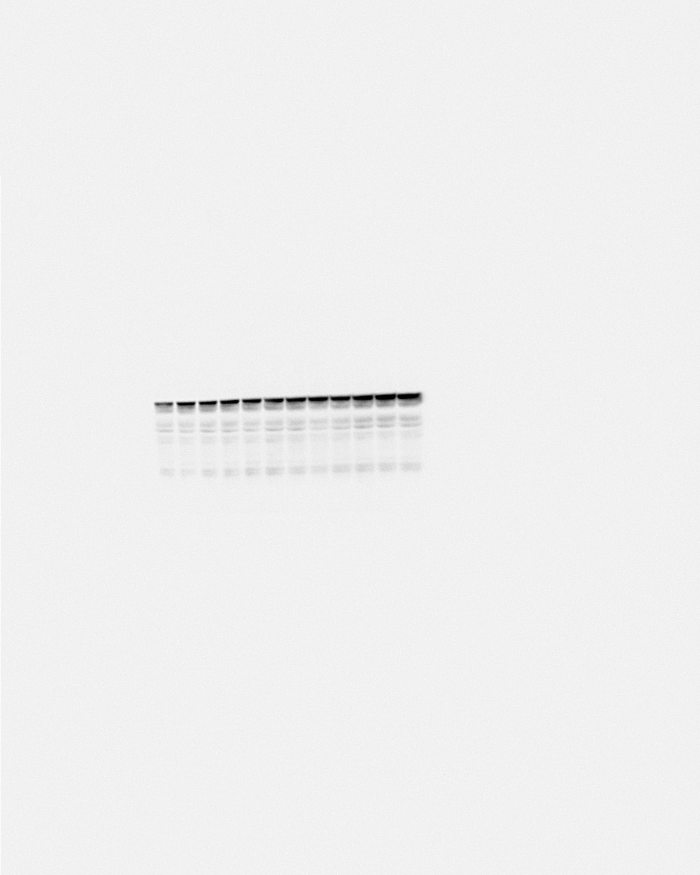

Supplement: Supplementary file 1 [file DataSheet1.zip › original-imagesú¿╤╣╦⌡║≤ú⌐/human blot/1/S100A8 (control 1-6+EACC 1-6).tif]

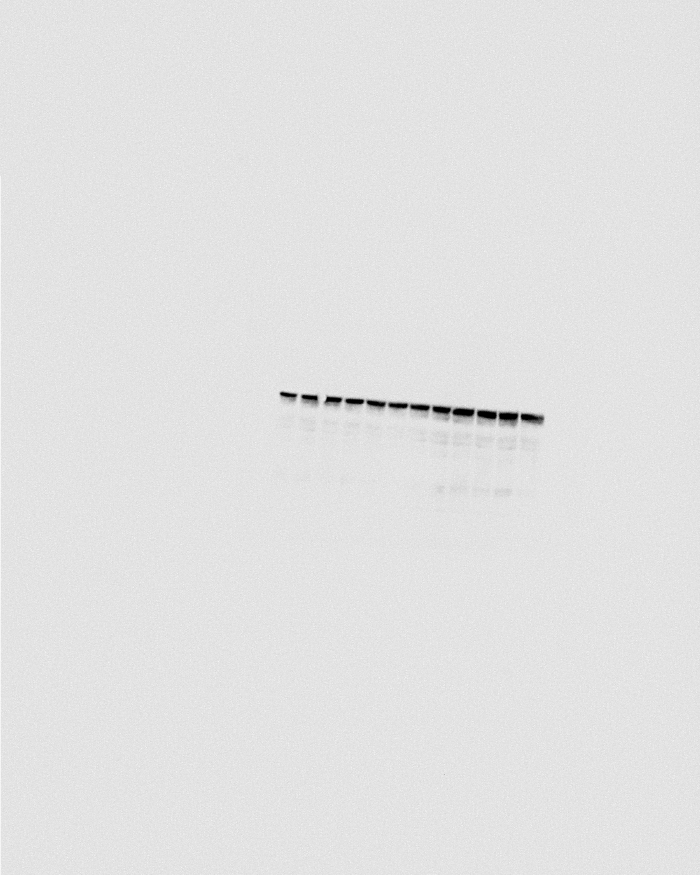

Supplement: Supplementary file 1 [file DataSheet1.zip › original-imagesú¿╤╣╦⌡║≤ú⌐/human blot/1/S100A8 (control 7-12+EACC 7-12).tif]

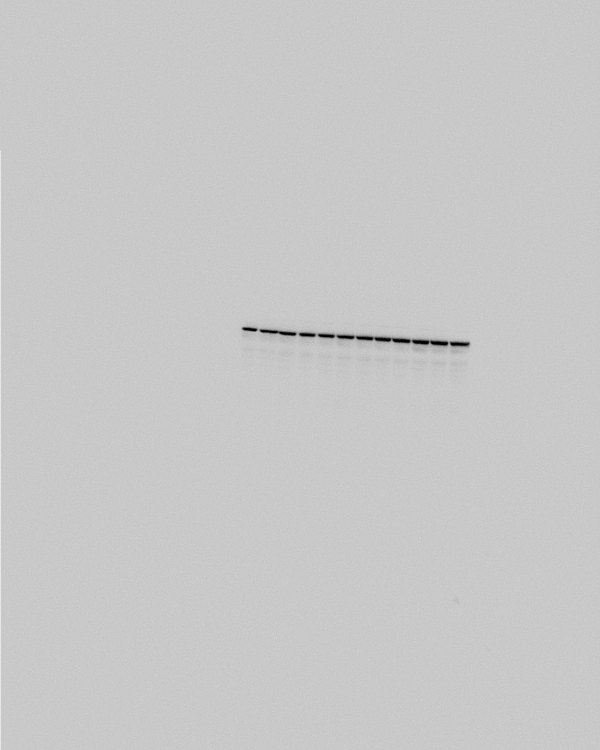

Supplement: Supplementary file 1 [file DataSheet1.zip › original-imagesú¿╤╣╦⌡║≤ú⌐/human blot/1/S100A9 (control 7-12+EACC 7-12).tif]

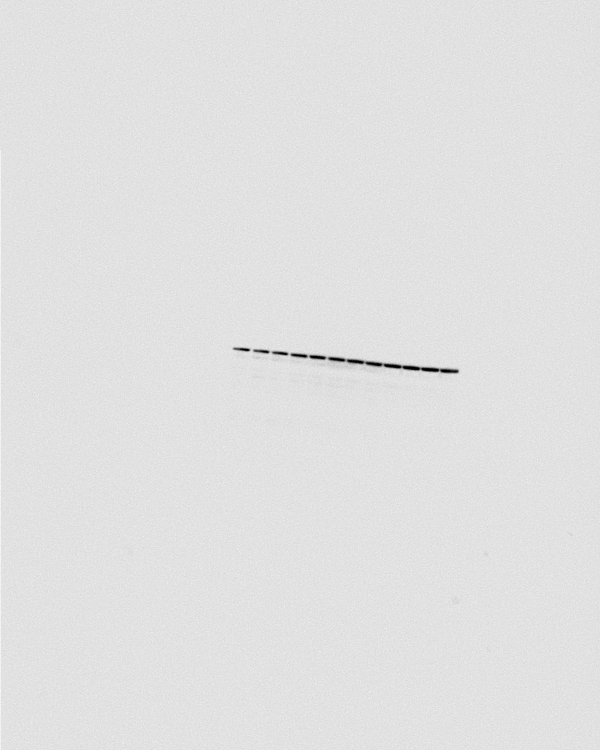

Supplement: Supplementary file 1 [file DataSheet1.zip › original-imagesú¿╤╣╦⌡║≤ú⌐/human blot/1/S100A9(control 1-6+EACC 1-6).tif]

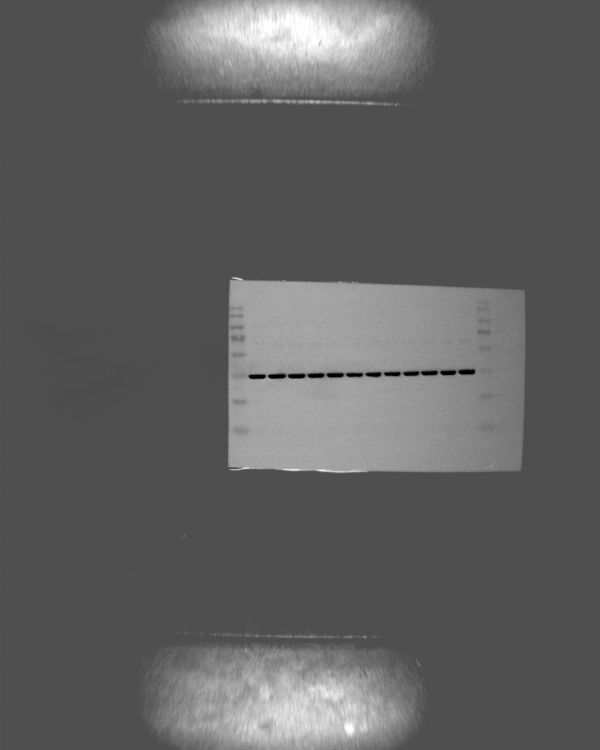

Supplement: Supplementary file 1 [file DataSheet1.zip › original-imagesú¿╤╣╦⌡║≤ú⌐/human blot/2/actin (control 1-6+EACC 1-6).tif]

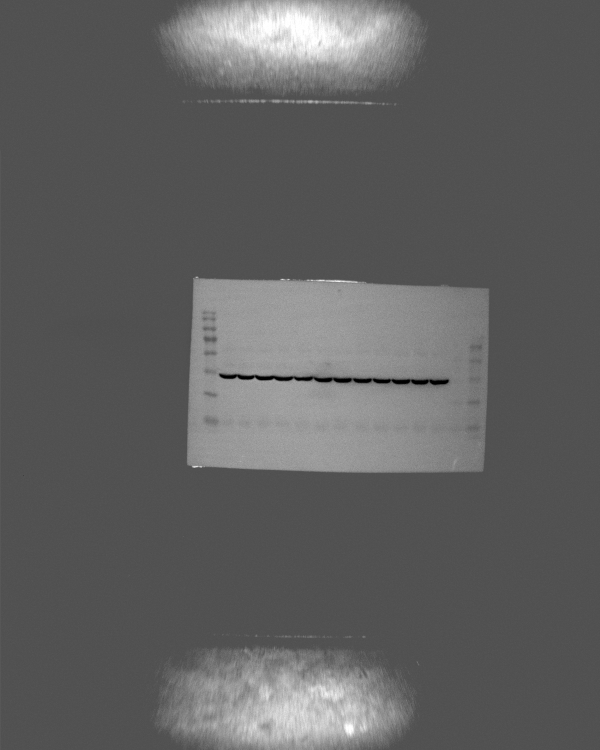

Supplement: Supplementary file 1 [file DataSheet1.zip › original-imagesú¿╤╣╦⌡║≤ú⌐/human blot/2/actin (control 7-12+EACC 7-12).tif]

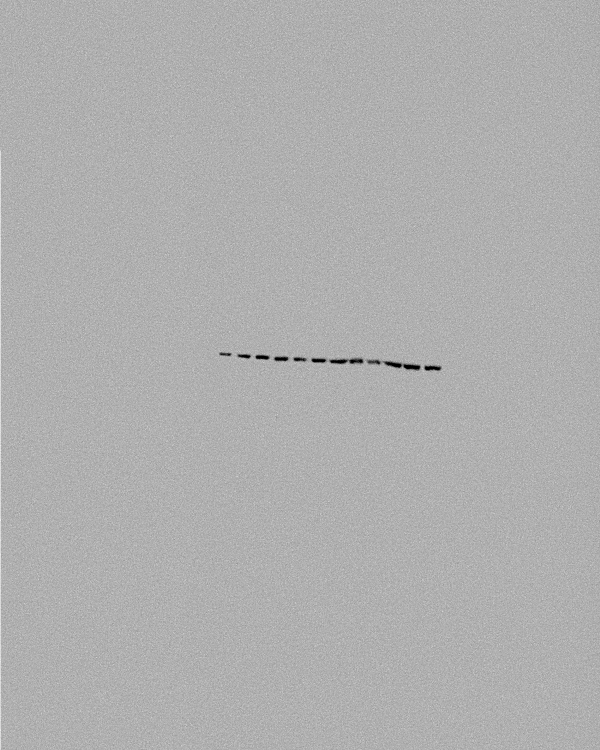

Supplement: Supplementary file 1 [file DataSheet1.zip › original-imagesú¿╤╣╦⌡║≤ú⌐/human blot/2/c-Met (control 7-12+EACC 7-12).tif]

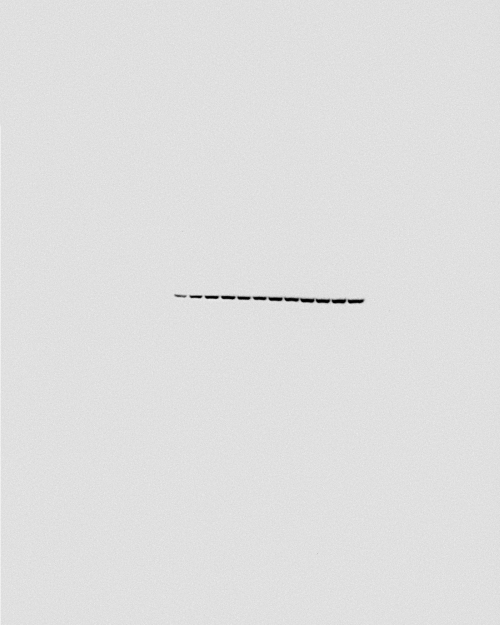

Supplement: Supplementary file 1 [file DataSheet1.zip › original-imagesú¿╤╣╦⌡║≤ú⌐/human blot/2/c-Met(control 1-6+EACC 1-6).tif]

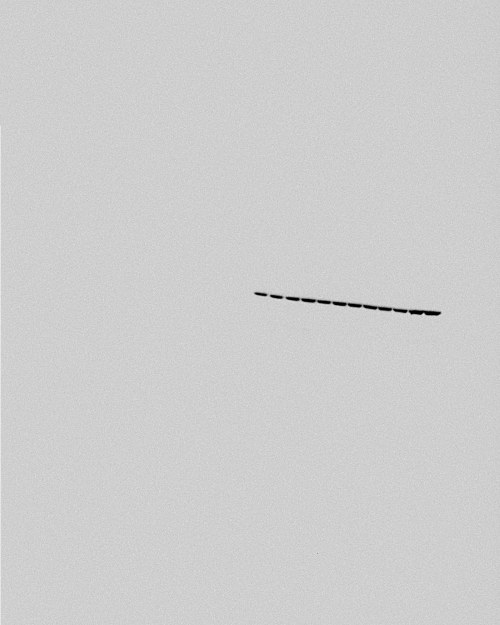

Supplement: Supplementary file 1 [file DataSheet1.zip › original-imagesú¿╤╣╦⌡║≤ú⌐/human blot/2/HGF SF (control 7-12+EACC 7-12).tif]

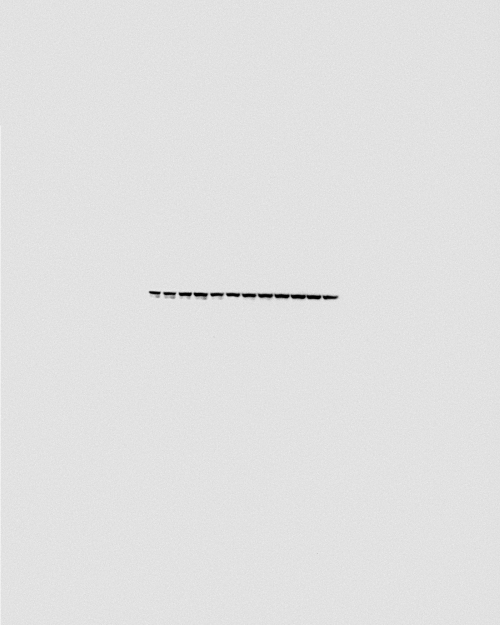

Supplement: Supplementary file 1 [file DataSheet1.zip › original-imagesú¿╤╣╦⌡║≤ú⌐/human blot/2/HGF SF(control 1-6+EACC 1-6).tif]

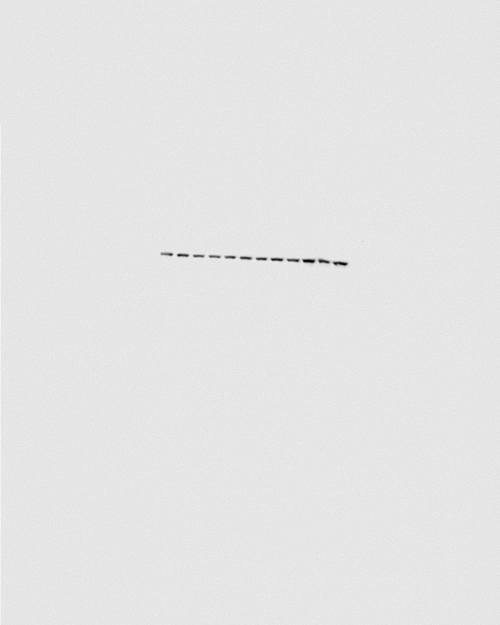

Supplement: Supplementary file 1 [file DataSheet1.zip › original-imagesú¿╤╣╦⌡║≤ú⌐/human blot/2/VEGF (control 1-6+EACC 1-6).tif]

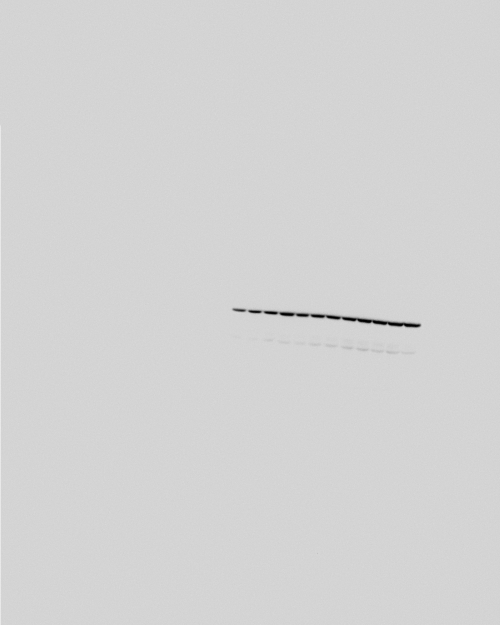

Supplement: Supplementary file 1 [file DataSheet1.zip › original-imagesú¿╤╣╦⌡║≤ú⌐/human blot/2/VEGF (control 7-12+EACC 7-12).tif]

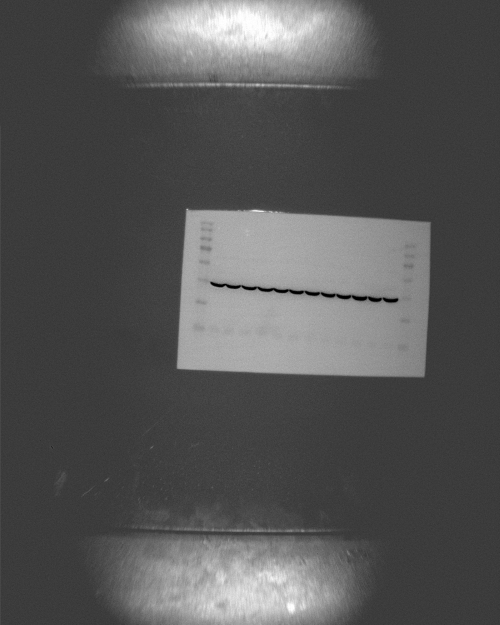

Supplement: Supplementary file 1 [file DataSheet1.zip › original-imagesú¿╤╣╦⌡║≤ú⌐/human blot/3/actin (control 1-6+EACC 1-6).tif]

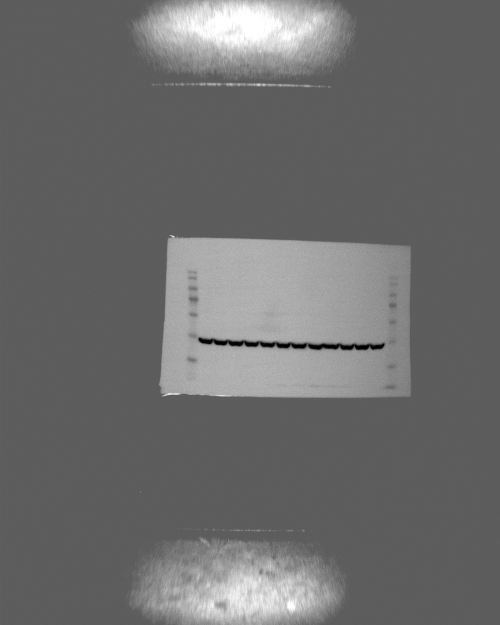

Supplement: Supplementary file 1 [file DataSheet1.zip › original-imagesú¿╤╣╦⌡║≤ú⌐/human blot/3/actin (control 7-12+EACC 7-12).tif]

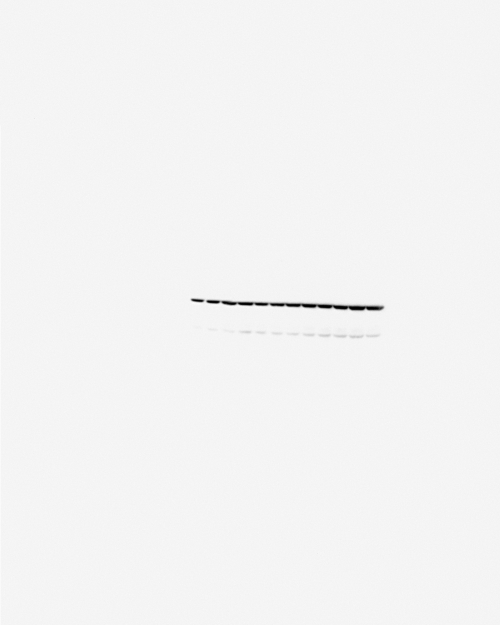

Supplement: Supplementary file 1 [file DataSheet1.zip › original-imagesú¿╤╣╦⌡║≤ú⌐/human blot/3/IFN-a├ (control 1-6+EACC 1-6).tif]

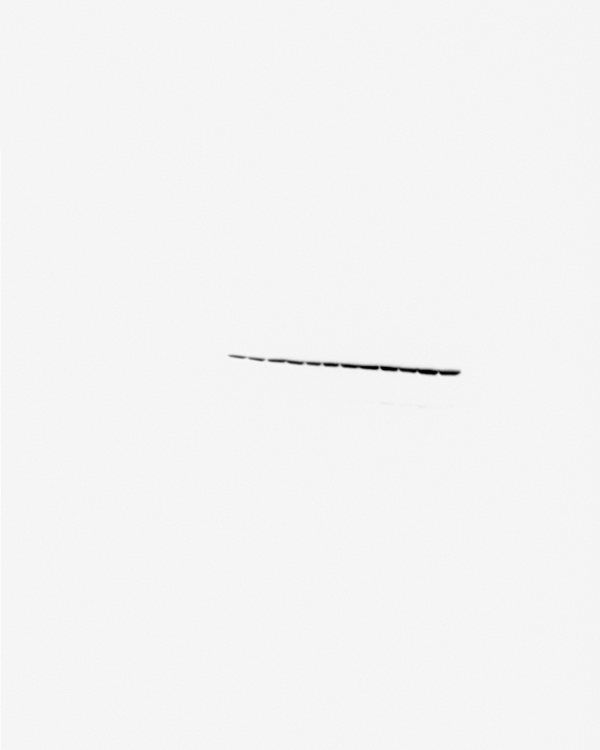

Supplement: Supplementary file 1 [file DataSheet1.zip › original-imagesú¿╤╣╦⌡║≤ú⌐/human blot/3/IFN-a├ (control 7-12+EACC 7-12).tif]

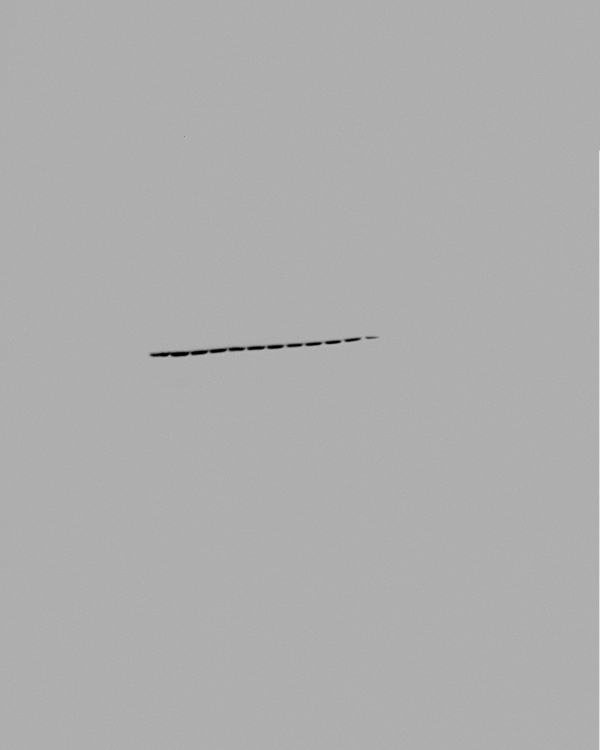

Supplement: Supplementary file 1 [file DataSheet1.zip › original-imagesú¿╤╣╦⌡║≤ú⌐/human blot/3/il-10 (control 1-6+EACC 1-6).tif]

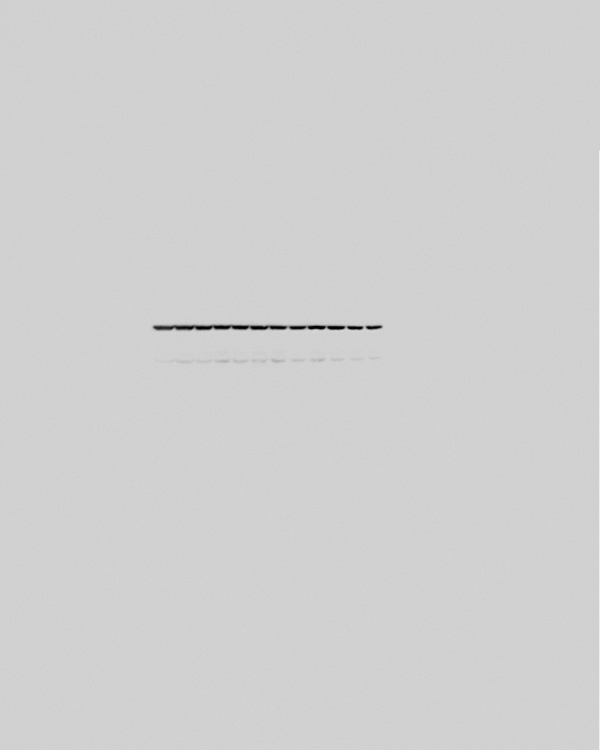

Supplement: Supplementary file 1 [file DataSheet1.zip › original-imagesú¿╤╣╦⌡║≤ú⌐/human blot/3/il-10 (control 7-12+EACC 7-12).tif]

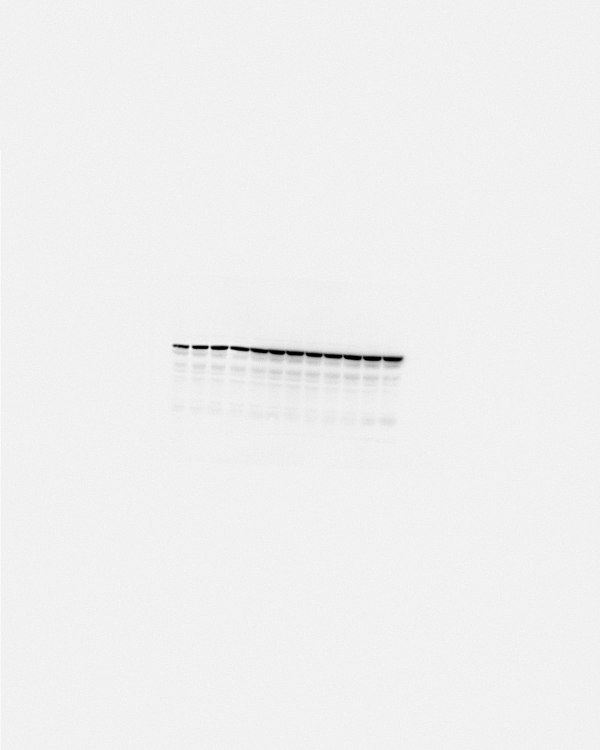

Supplement: Supplementary file 1 [file DataSheet1.zip › original-imagesú¿╤╣╦⌡║≤ú⌐/human blot/3/TGF-a┬ (control 1-6+EACC 1-6).tif]

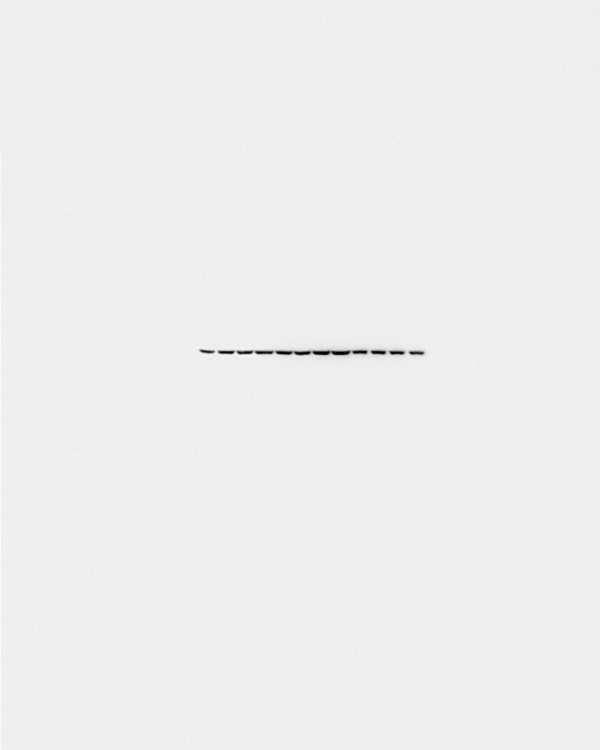

Supplement: Supplementary file 1 [file DataSheet1.zip › original-imagesú¿╤╣╦⌡║≤ú⌐/human blot/3/TGF-a┬ (control 7-12+EACC 7-12).tif]

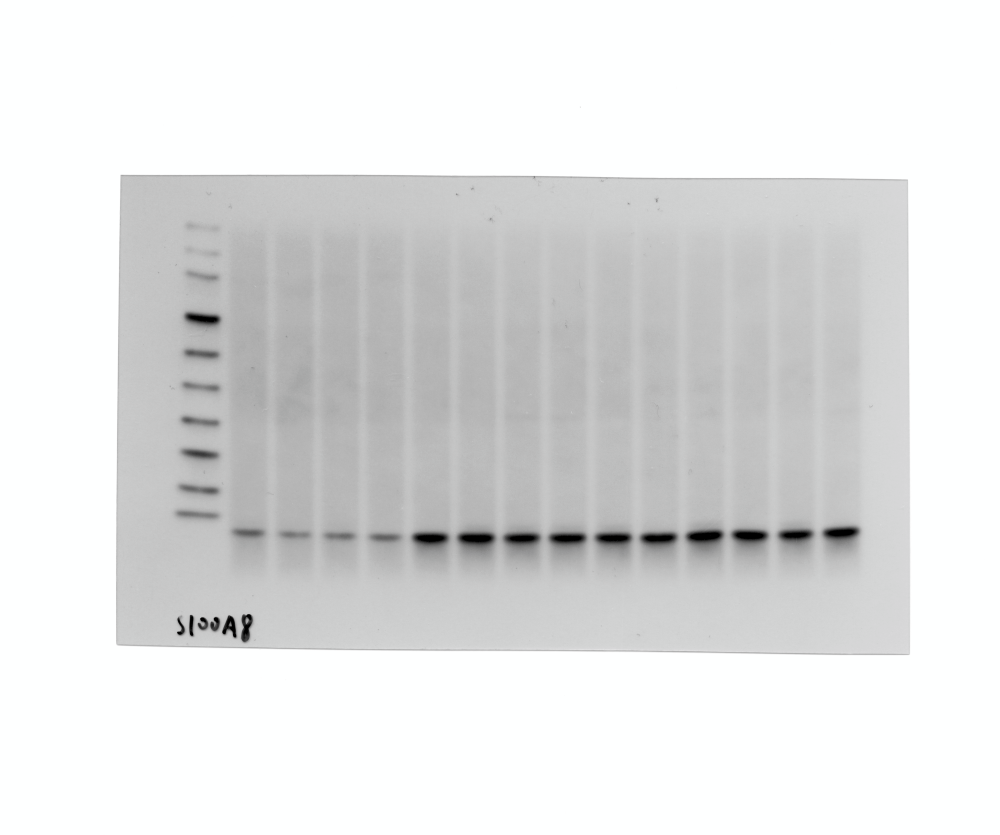

Supplement: Supplementary file 1 [file DataSheet1.zip › original-imagesú¿╤╣╦⌡║≤ú⌐/mice blot/1/S100A8(control 4+EACC 10).tif]

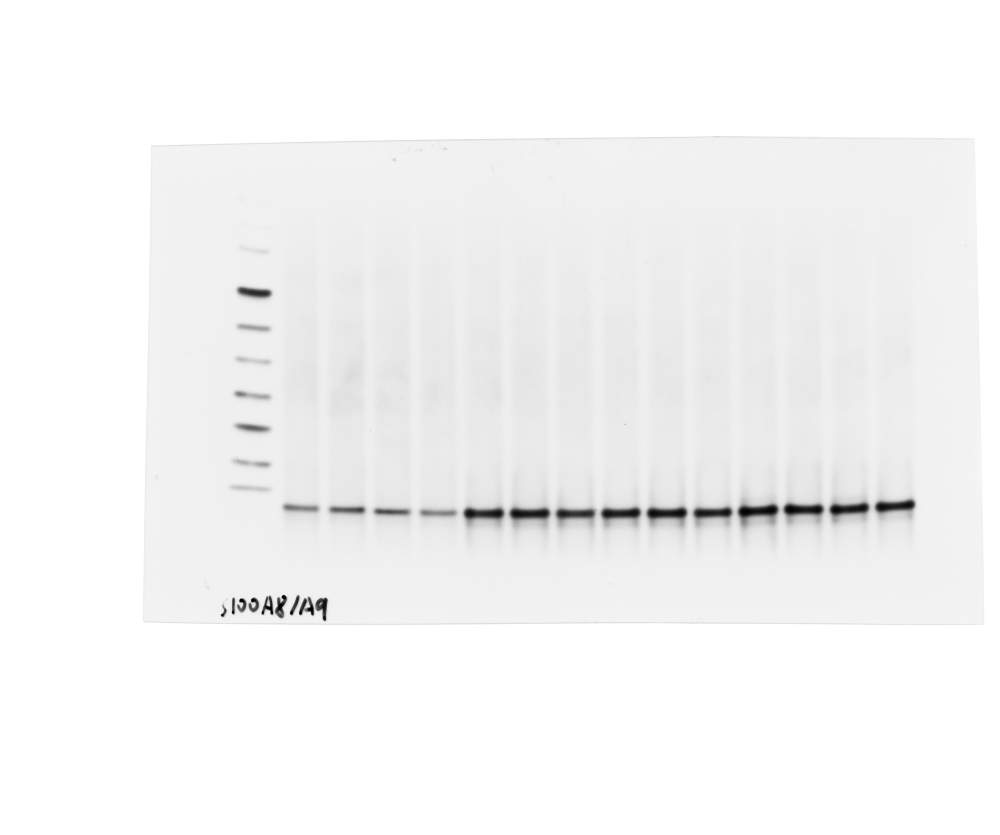

Supplement: Supplementary file 1 [file DataSheet1.zip › original-imagesú¿╤╣╦⌡║≤ú⌐/mice blot/1/S100A8A9(control 4+EACC 10).tif]

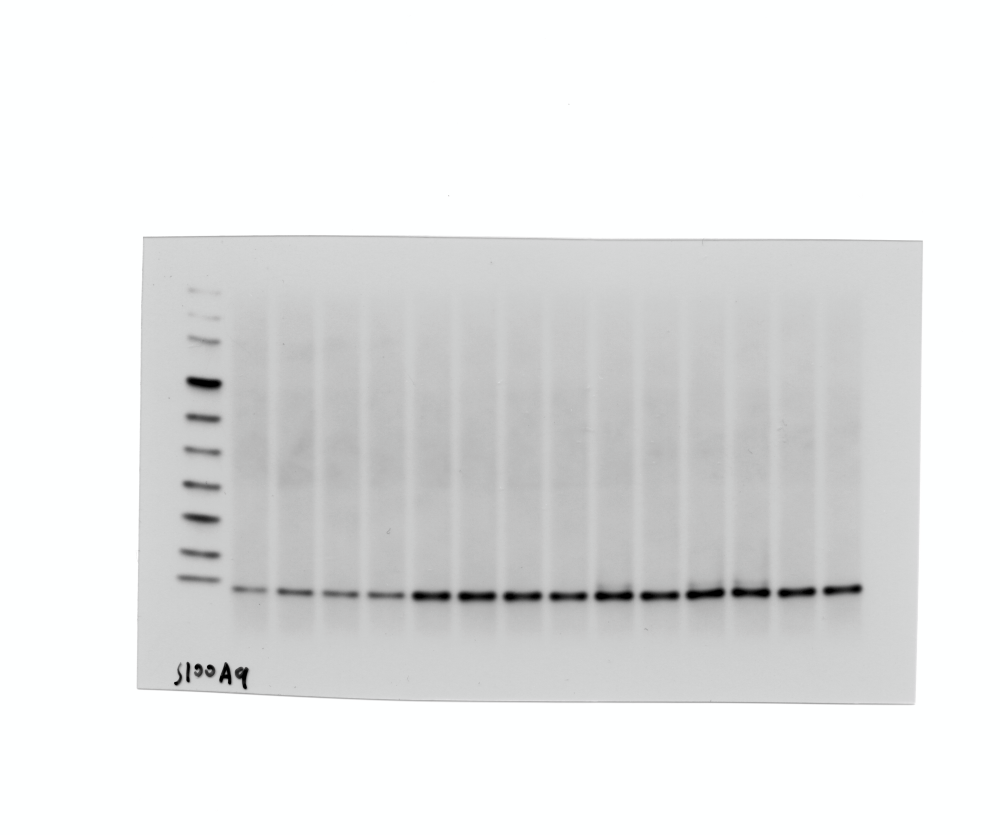

Supplement: Supplementary file 1 [file DataSheet1.zip › original-imagesú¿╤╣╦⌡║≤ú⌐/mice blot/1/S100A9(control 4+EACC 10).tif]

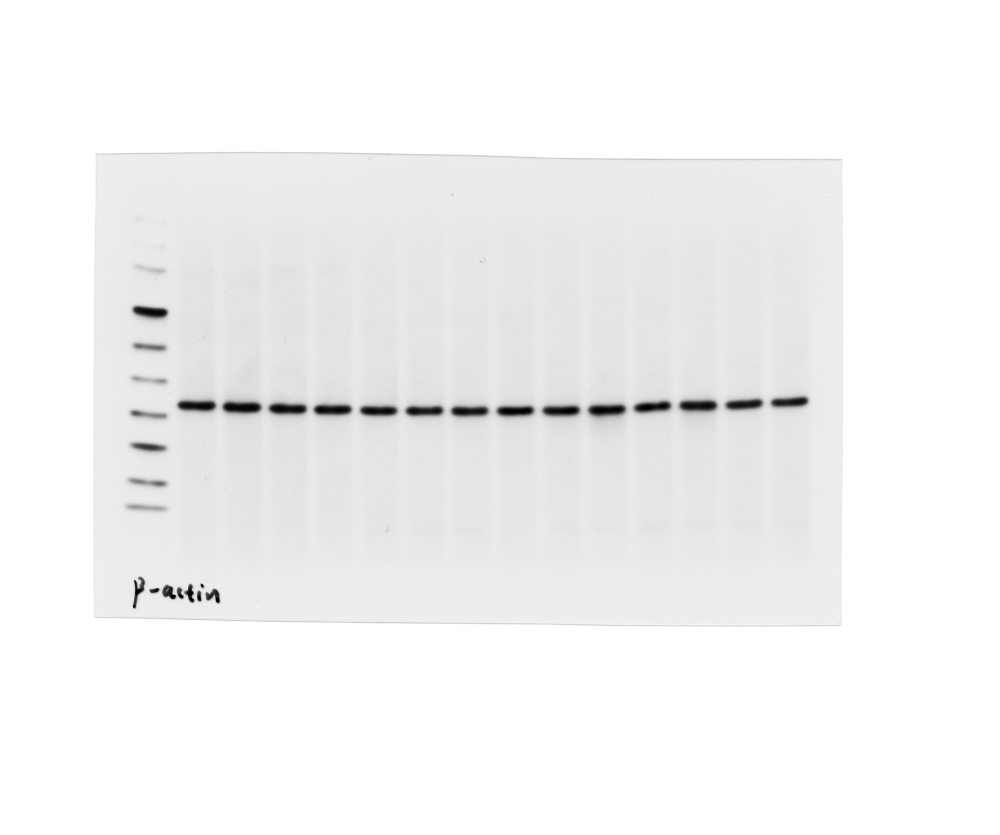

Supplement: Supplementary file 1 [file DataSheet1.zip › original-imagesú¿╤╣╦⌡║≤ú⌐/mice blot/1/a┬-Actin.tif]

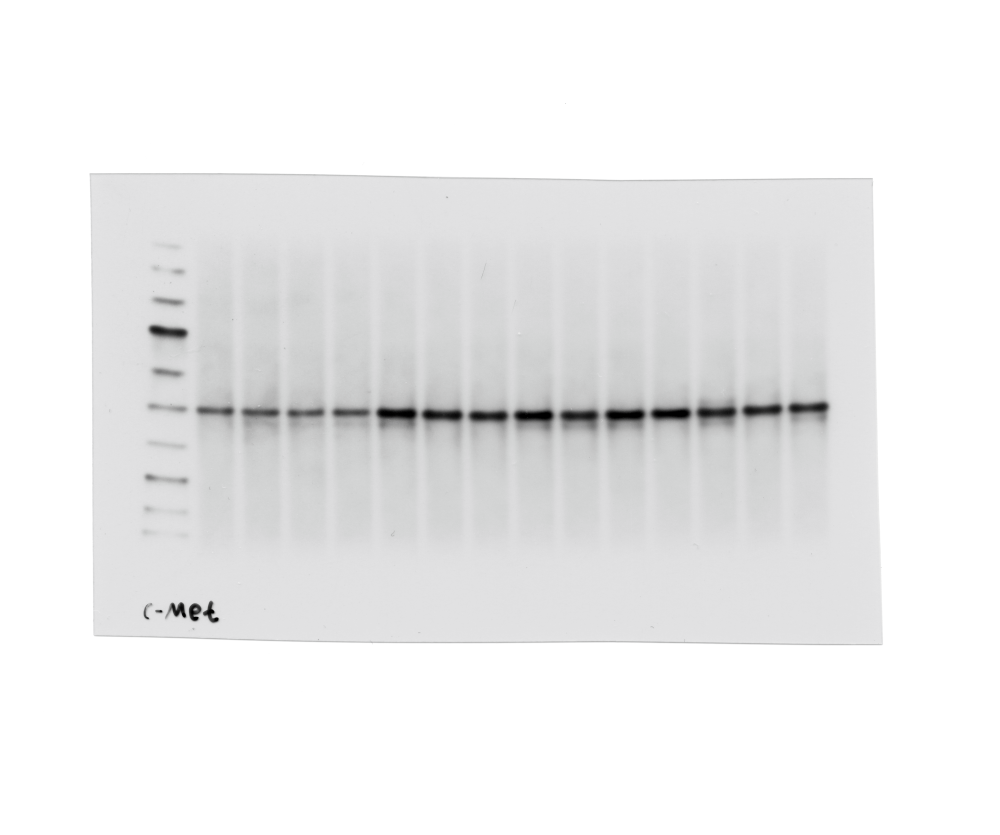

Supplement: Supplementary file 1 [file DataSheet1.zip › original-imagesú¿╤╣╦⌡║≤ú⌐/mice blot/2/c-Met(control 4+EACC 10).tif]

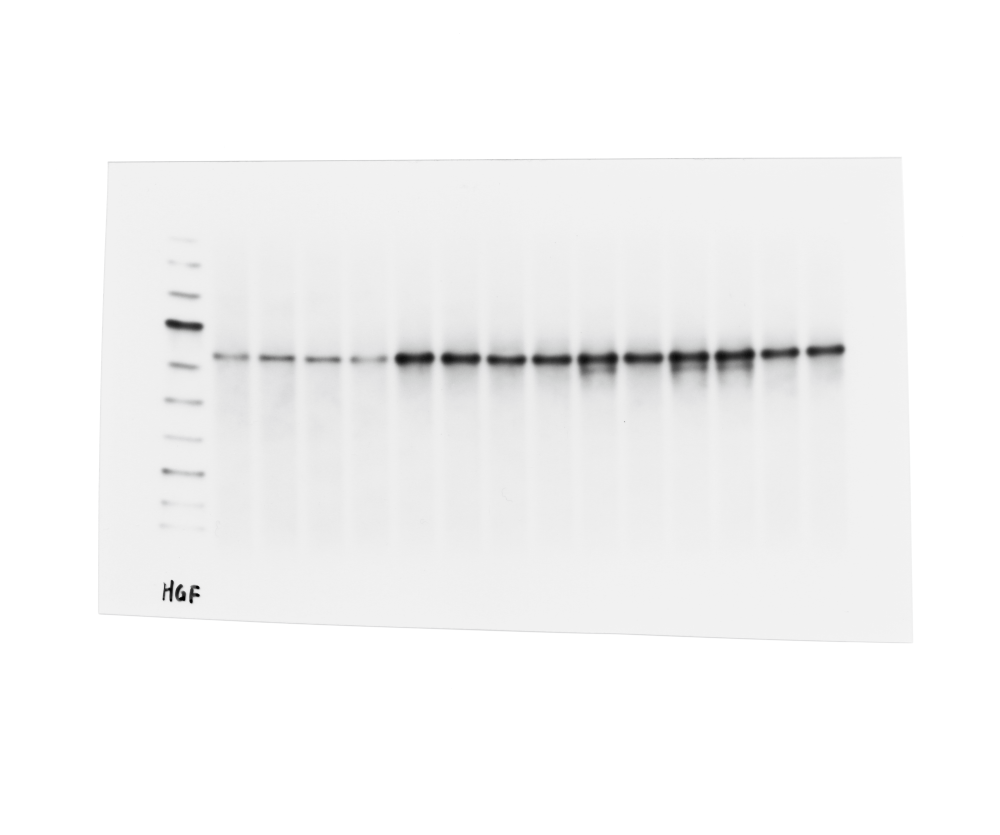

Supplement: Supplementary file 1 [file DataSheet1.zip › original-imagesú¿╤╣╦⌡║≤ú⌐/mice blot/2/HGF(control 4+EACC 10).tif]

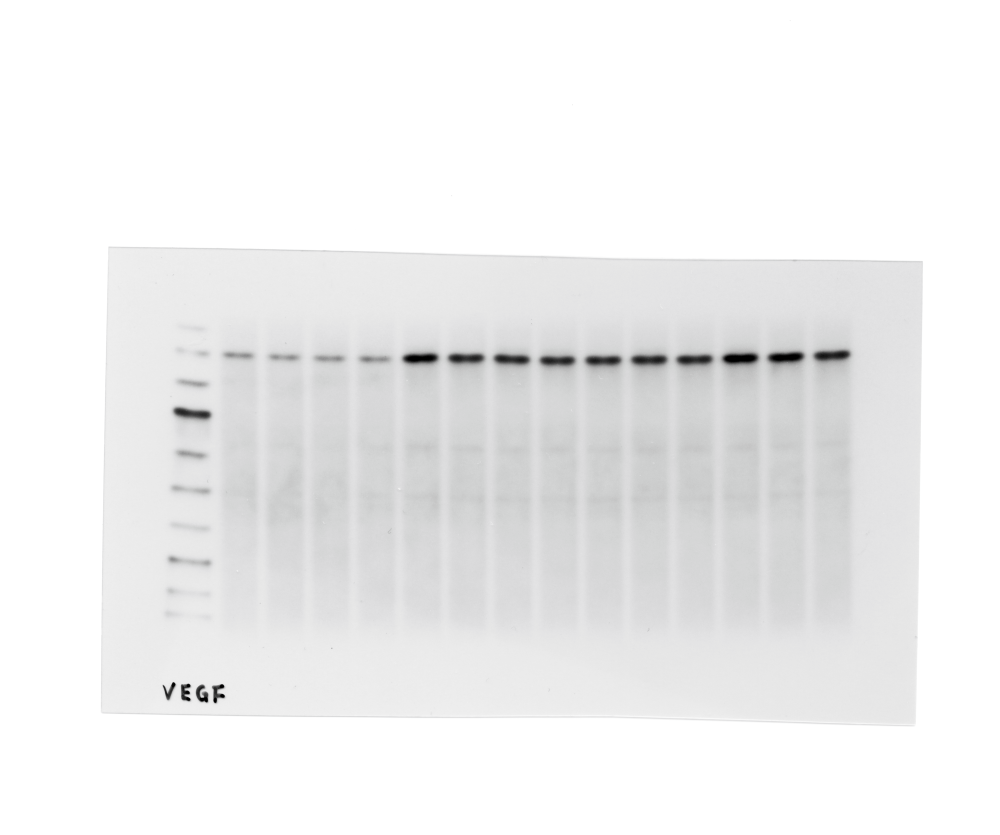

Supplement: Supplementary file 1 [file DataSheet1.zip › original-imagesú¿╤╣╦⌡║≤ú⌐/mice blot/2/VEGF(control 4+EACC 10).tif]

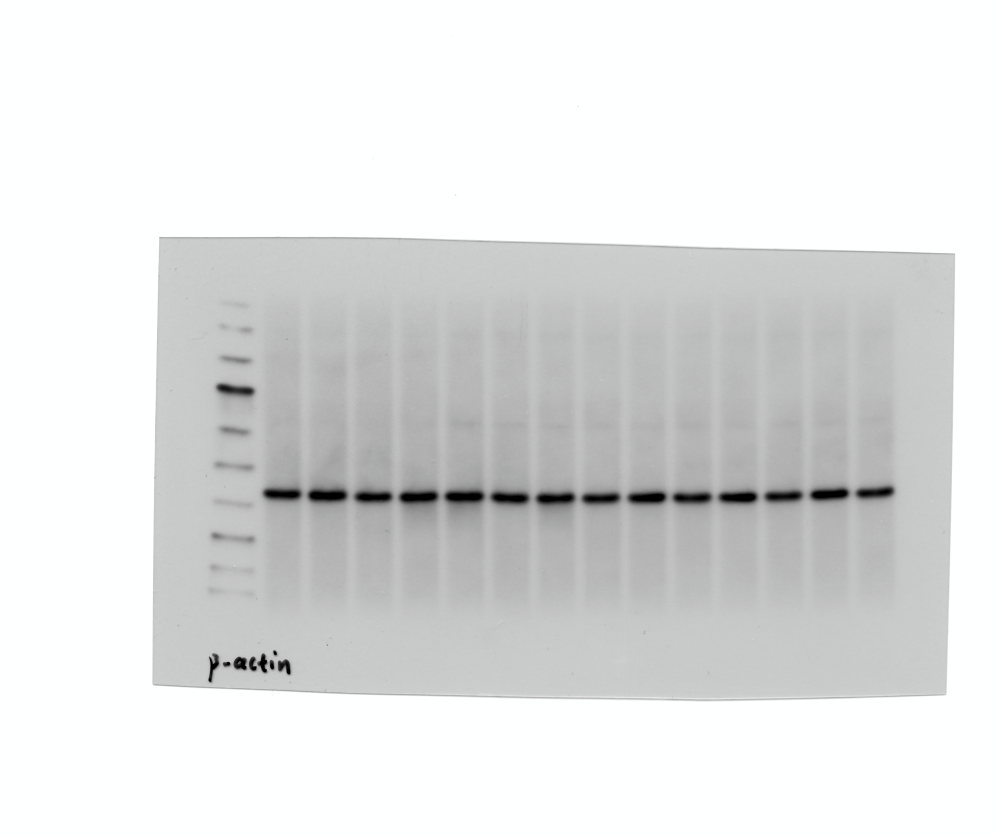

Supplement: Supplementary file 1 [file DataSheet1.zip › original-imagesú¿╤╣╦⌡║≤ú⌐/mice blot/2/a┬-Actin.tif]

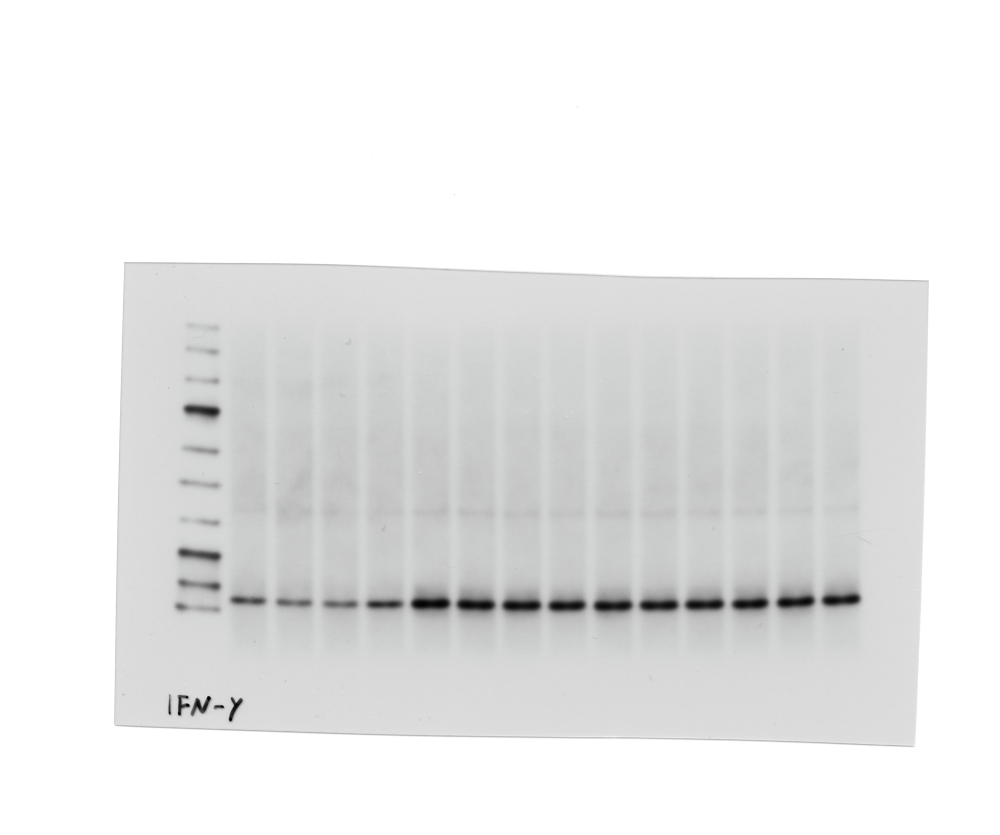

Supplement: Supplementary file 1 [file DataSheet1.zip › original-imagesú¿╤╣╦⌡║≤ú⌐/mice blot/3/IFN-a├(control 4+EACC 10).tif]

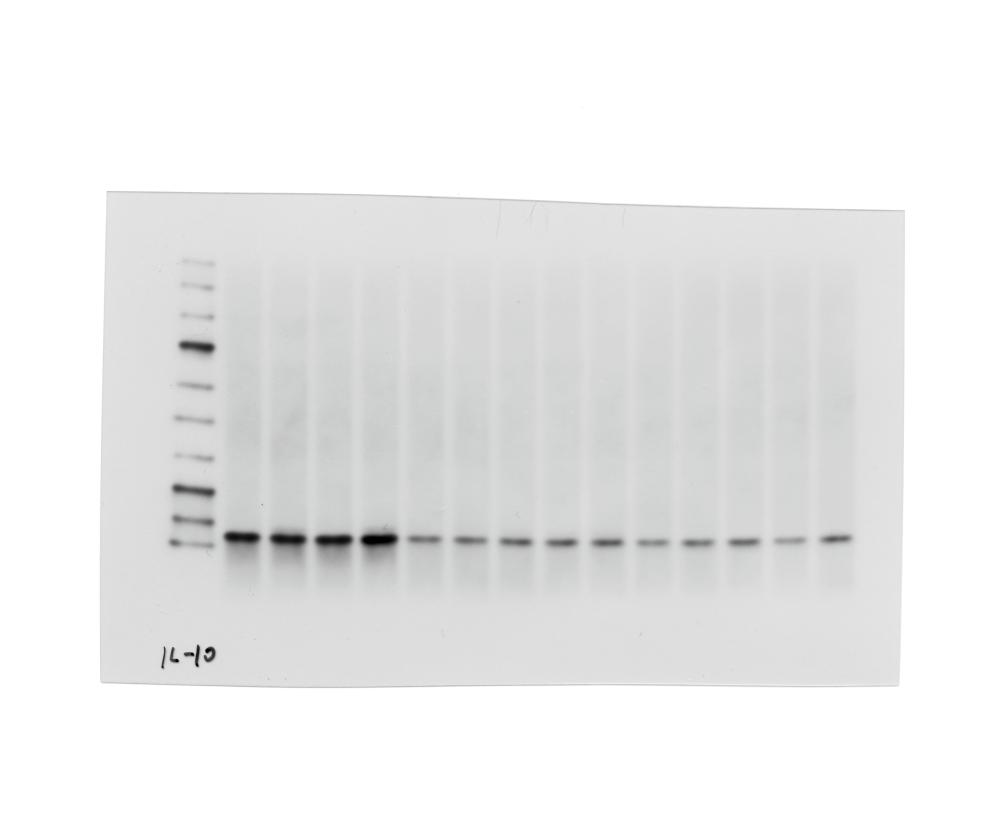

Supplement: Supplementary file 1 [file DataSheet1.zip › original-imagesú¿╤╣╦⌡║≤ú⌐/mice blot/3/IL-10(control 4+EACC 10).tif]

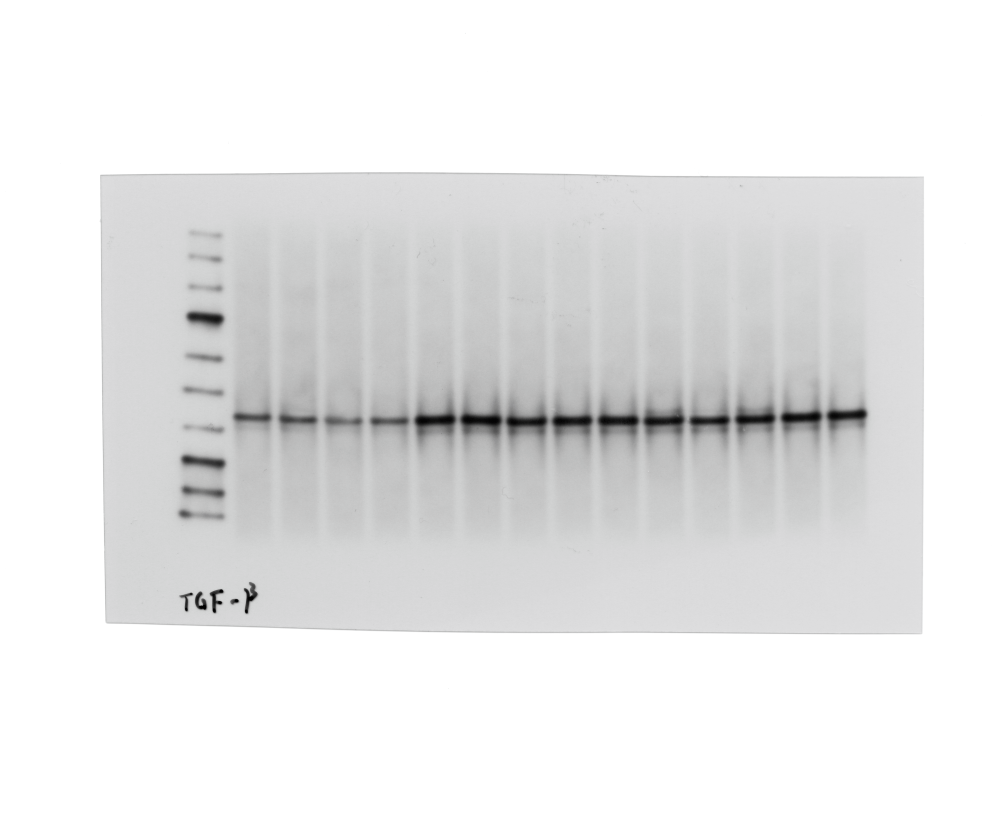

Supplement: Supplementary file 1 [file DataSheet1.zip › original-imagesú¿╤╣╦⌡║≤ú⌐/mice blot/3/TGF-a┬(control 4+EACC 10).tif]

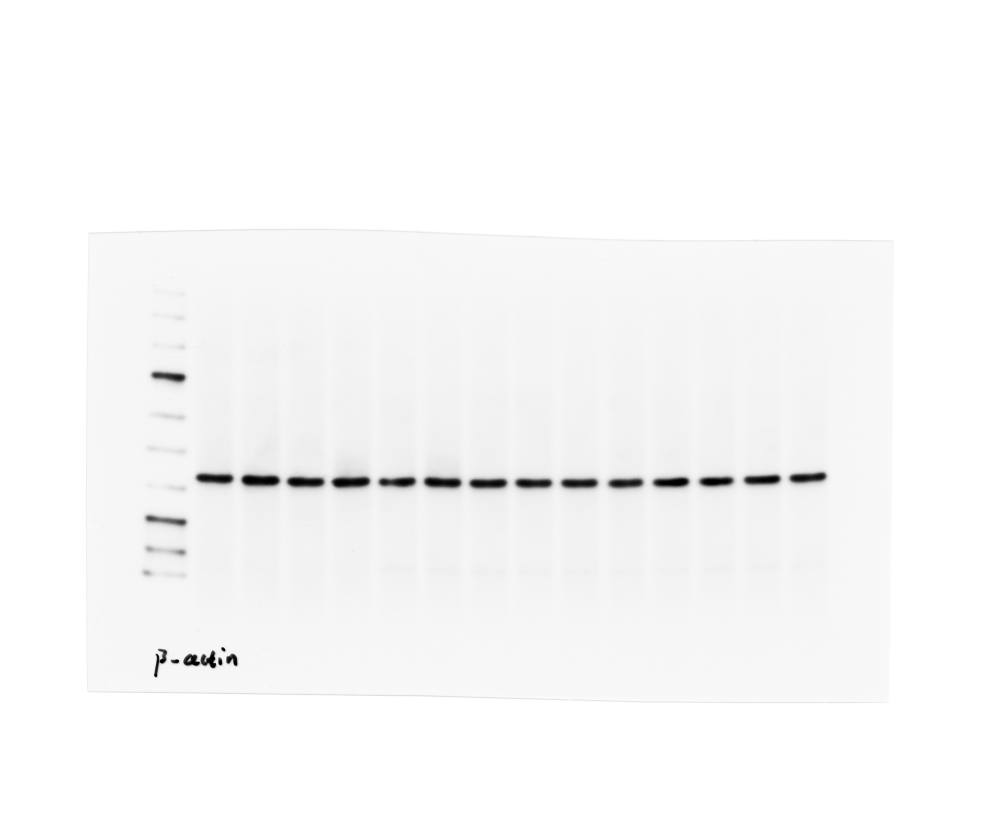

Supplement: Supplementary file 1 [file DataSheet1.zip › original-imagesú¿╤╣╦⌡║≤ú⌐/mice blot/3/a┬-Actin.tif]
